# Impact of ambient air pollution on colorectal cancer risk and survival: insights from a prospective cohort and epigenetic Mendelian randomization study

**DOI:** 10.1016/j.ebiom.2024.105126

**Published:** 2024-04-16

**Authors:** Fangyuan Jiang, Jianhui Zhao, Jing Sun, Wenxi Chen, Yuyuan Zhao, Siyun Zhou, Shuai Yuan, Maria Timofeeva, Philip J. Law, Susanna C. Larsson, Dong Chen, Richard S. Houlston, Malcolm G. Dunlop, Evropi Theodoratou, Xue Li

**Affiliations:** a Department of Big Data in Health Science, School of Public Health and the Second Affiliated Hospital, Zhejiang University School of Medicine, Hangzhou, China; b Unit of Cardiovascular and Nutritional Epidemiology, Institute of Environmental Medicine, Karolinska Institute, Stockholm, Sweden; c Danish Institute for Advanced Study (DIAS), Epidemiology, Biostatistics and Biodemography Research Unit, Institute of Public Health, University of Southern Denmark, Odense, Denmark; d Division of Genetics and Epidemiology, Institute of Cancer Research, London, UK; e Unit of Medical Epidemiology, Department of Surgical Sciences, Uppsala, Sweden; f Department of Colorectal Surgery, The First Affiliated Hospital, Zhejiang University School of Medicine, Hangzhou, 310003, Zhejiang Province, China; g Cancer Research UK Edinburgh Centre, Medical Research Council Institute of Genetics and Cancer, University of Edinburgh, Edinburgh, UK; h Centre for Global Health, Usher Institute, The University of Edinburgh, Edinburgh, UK

**Keywords:** Air pollution, Colorectal cancer, Incidence, Survival, Mendelian, Randomization, DNA methylation

## Abstract

**Background:**

This study investigates the associations between air pollution and colorectal cancer (CRC) risk and survival from an epigenomic perspective.

**Methods:**

Using a newly developed Air Pollutants Exposure Score (APES), we utilized a prospective cohort study (UK Biobank) to investigate the associations of individual and combined air pollution exposures with CRC incidence and survival, followed by an up-to-date systematic review with meta-analysis to verify the associations. In epigenetic two-sample Mendelian randomization analyses, we examine the associations between genetically predicted DNA methylation related to air pollution and CRC risk. Further genetic colocalization and gene–environment interaction analyses provided different insights to disentangle pathogenic effects of air pollution via epigenetic modification.

**Findings:**

During a median 12.97-year follow-up, 5767 incident CRC cases among 428,632 participants free of baseline CRC and 533 deaths in 2401 patients with CRC were documented in the UK Biobank. A higher APES score was associated with an increased CRC risk (HR, 1.03, 95% CI = 1.01–1.06; *P* = 0.016) and poorer survival (HR, 1.13, 95% CI = 1.03–1.23; *P* = 0.010), particularly among participants with insufficient physical activity and ever smokers (P_interaction_ > 0.05). A subsequent meta-analysis of seven observational studies, including UK Biobank data, corroborated the association between PM_2.5_ exposure (per 10 μg/m^3^ increment) and elevated CRC risk (RR,1.42, 95% CI = 1.12–1.79; *P* = 0.004; I^2^ = 90.8%). Genetically predicted methylation at PM_2.5_-related CpG site cg13835894 near *TMBIM1/PNKD* and cg16235962 near *CXCR5*, and NO_2_-related cg16947394 near *TMEM110* were associated with an increased CRC risk. Gene-environment interaction analysis confirmed the epigenetic modification of aforementioned CpG sites with CRC risk and survival.

**Interpretation:**

Our study suggests the association between air pollution and CRC incidence and survival, underscoring the possible modifying roles of epigenomic factors. Methylation may partly mediate pathogenic effects of air pollution on CRC, with annotation to epigenetic alterations in protein-coding genes *TMBIM1/PNKD*, *CXCR5* and *TMEM110*.

**Funding:**

Xue Li is supported by the Natural Science Fund for Distinguished Young Scholars of Zhejiang Province (LR22H260001), the National Nature Science Foundation of China (No. 82204019) and Healthy Zhejiang One Million People Cohort (K-20230085). ET is supported by a Cancer Research UK Career Development Fellowship (C31250/A22804). MGD is supported by the MRC Human Genetics Unit Centre Grant (U127527198).

**Research in context**

**Evidence before this study**

Sporadic and inconsistent epidemiological evidence has linked air pollution to the development of colorectal cancer (CRC). The epigenome is emerging as a vital link between environmental exposure, alterations in gene expression, and the onset of CRC. Whether and how air pollution plays a role in the development of CRC was inconclusive and needed to be clarified.

**Added value of this study**

We initially introduced the Air Pollutants Exposure Score (APES) and unveiled that a higher APES score was associated with both an increased CRC risk and poorer survival, specifically among participants with insufficient physical activity and ever smokers. The epigenetic MR analyses disclosed that genetically predicted methylation at two PM_2.5_- and one NO_2_-related CpG site appeared to elevate CRC risk. And the impact of epigenetic modifications on CRC risk and survival in protein-coding genes *TMBIM1/PNKD*, *CXCR5*, and *TMEM110* were additionally validated.

**Implications of all the available evidence**

Leveraging the integrative observational, genetic, and methylation evidence, our study underscored the crucial importance of assessing combined air pollution effects and other modifiable environmental factors for both primary and secondary CRC prevention. It also shed light on the profound impact of epigenetic alterations in the complex pathogenesis of CRC, suggesting that methylation may serve as partial mediators in the deleterious effects of air pollution on CRC.

## Introduction

Colorectal cancer (CRC) is one of the most common cancers as well as a leading cause of cancer-related death worldwide.^1^ Its etiology encompasses a spectrum of lifestyle and environmental factors.^2^^,^^3^ As the most significant environmental health threat,^4^ ambient air pollution has been associated with cancer development through affecting the inflammatory system.^5^^,^^6^ Sporadic and inconsistent epidemiological evidence has linked air pollution to the development of CRC.^7^, ^8^, ^9^, ^10^ A recent meta-analysis indicated significant associations between particulate matter (PM) and an evaluated risk of gastrointestinal cancer incidence and mortality, notably liver cancer and CRC.^11^ The specific role of PM in CRC incidence remains inconclusive,^7^^,^^8^ with the effects of other pollutants (such as nitrogen oxides) and their combined impacts being largely unexplored. Similarly, while research has recognized detrimental effects of long-term air pollution exposure on the mortality risk among patients diagnosed with cancer,^12^^,^^13^ insights into overall survival in patients with CRC are lacking.

Although air pollution has been shown to affect the immune system and is associated with chronic systemic inflammation, oxidative stress, and DNA damage during cancer development,^5^^,^^14^ the underlying mechanisms remain inadequately derdetermined. Emerging epigenome-wide association studies (EWAS) suggested that air pollution exposure altered epigenetic marks, specifically DNA methylation (DNAm),^15^, ^16^, ^17^ which might in turn influence inflammation, disease development, and exacerbation risk.^18^ Meanwhile, the aberrant DNAm patterns, characterized by 5-methylcytosine formation in cytosine-phosphate-guanine (CpG) dinucleotides, have been identified as a crucial epigenetic mechanism in CRC carcinogenesis by hindering the combination of the transcription complex and DNA, resulting in unprogrammed alterations in downstream gene expression.^19^^,^^20^ However, how air pollution modifies DNAm and subsequently contributes to the pathological effects on CRC is unknown.

Mendelian randomization (MR) analysis is an effective tool for causal inference by utilizing genetic variants as proxies for an exposure (e.g., air pollution-related DNAm), which thus minimizes confounding or reverse causality.^21^ In our study, we examined the associations of individual and combined ambient air pollution exposures (particulate matters including PM_10_ and PM_2.5_, and nitrogen oxides including NO_x_ and NO_2_) with CRC risk and overall survival. We further explored the potential pathological effect related to air pollution induced DNAm and the gene–environment interaction effects.

## Methods

### Study design

**Fig. 1:**
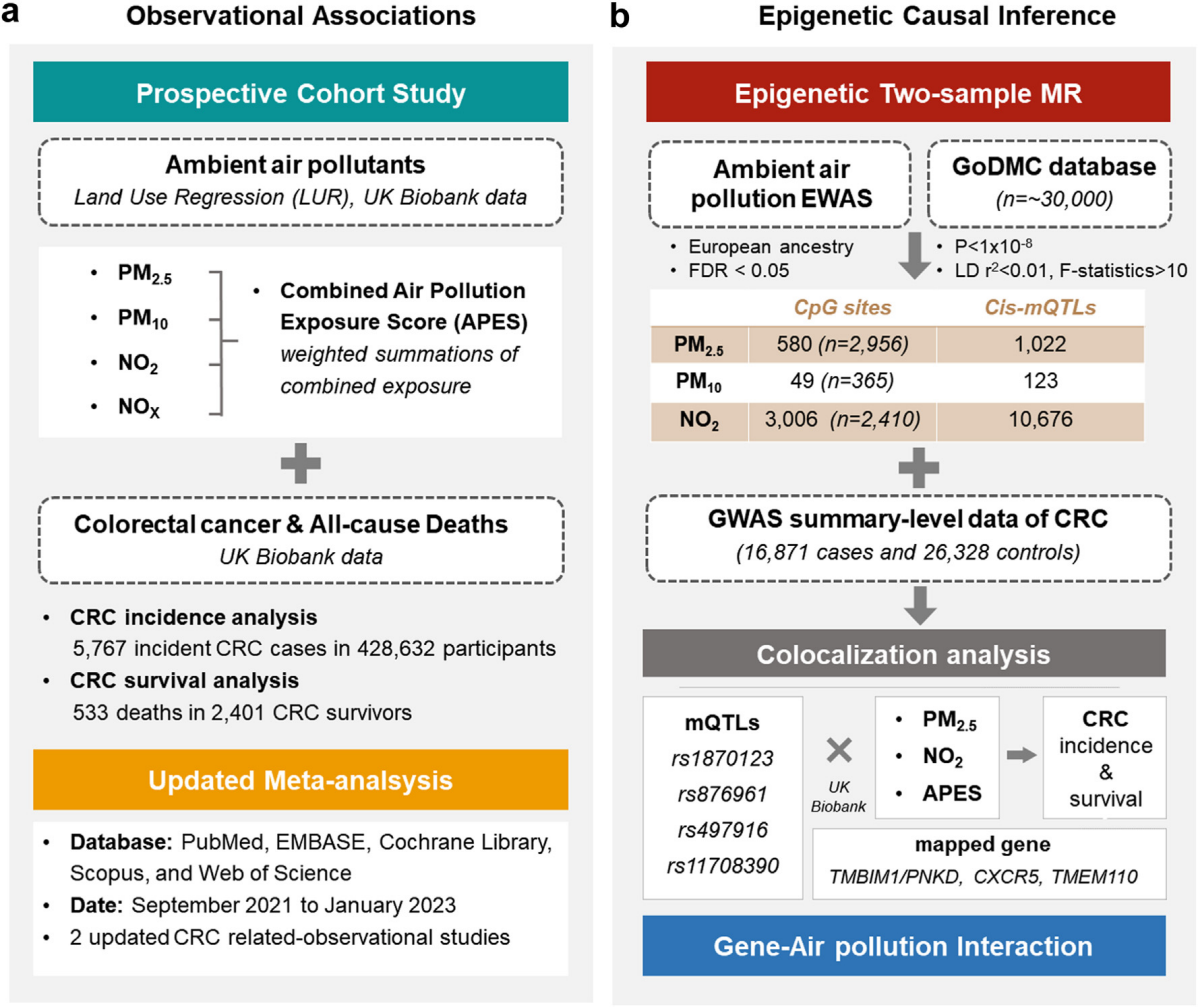
**Schematic diagram of the study design**.

Fig. 1 shows the study design overview. We initially investigated the associations between air pollution and both CRC incidence and survival among patients with CRC in the UK Biobank cohort. We subsequently conducted an up-to-date systematic review with meta-analysis investigating the association between PM exposure and CRC outcomes (incidence or CRC specific-mortality) to validate the epidemiologic associations. To elucidate the underlying pathogenic mechanism, two-sample MR analyses were utilized, incorporating genetic instruments for blood DNAm related to air pollution and summary-level Genome-Wide Association Study (GWAS) data for CRC incidence. In addition, genetic colocalization and gene–environment interaction analyses were performed to further shed light on the potential carcinogenic or prognostic effects of air pollution on CRC.

### Prospective cohort study and updated meta-analysis

#### Study population

The UK Biobank is a large-scale cohort study (∼500,000 participants aged 40–69 at enrollment) collecting genotypic and phenotypic health-related information. Participants with other cancer diagnoses at baseline (n = 34,969) and those lacking data on air pollution (n = 38,889) were removed from the CRC incidence analysis, resulting in a cohort of 428,632 participants for this analysis (Supplemental Figure S1). The evaluation of all-cause mortality among patients with CRC was confined to participants with prior CRC diagnosis at recruitment. After excluding those with missing air pollution data (n = 229), 2401 patients with CRC were eligible for analysis of which 533 died (Supplemental Figure S1). The UK Biobank received the ethical approval from the North West Multicenter Research Ethics Committee (REC reference: 21/NW/0157) and all participants signed consent.

#### Assessment of air pollutants

We treated four air pollutants (NO_x_, NO_2_, PM_10_, and PM_2.5_) as primary exposures based on data availability in the UK Biobank and corresponding criteria set by the World Health Organization (2021) and European Commission (2022). The annual average concentrations were modeled by Land Use Regression (LUR) techniques using the predictor variables obtained from the Geographic Information System and the linkage to participants’ residential addresses at the baseline visit.^22^ The LUR model demonstrated high performance for PM_2.5_, PM_10_, NO_2_, and NO_x_ with a cross-validation R^2^ of 77%, 88%, 87%, and 88%, respectively. For PM_2.5_ and NO_x_, air pollution estimates were only available for the year 2010. NO_2_ (2005-7 and 2010) and PM_10_ (2007 and 2010) had data for multiple years; hence, we employed the mean values in the analysis. To quantify the combined exposure to various air pollutants and its impact on CRC, a weighted air pollutants exposure score (APES) was created based on the β coefficient of each air pollutant in multivariable Cox regression: APES = (βPM_2.5_∗PM_2.5_ + βPM_10_∗PM_10_ + βNO_2_∗NO_2_ + βNO_x_∗NO_x_) ∗ (4/sum of the β coefficients)^23^ (details in Supplementary Table S1).

#### Ascertainment of incidence and deaths of CRC

The clinical outcomes of interest were incident CRC and all-cause mortality among CRC participants. CRC diagnosis was confirmed via linkage to national cancer registries in England, Wales, and Scotland (follow-up through 20 February 2022) and inpatient medical records (follow-up through 31 March 2021). The International Classification of Diseases codes (ICD-9: 153, 154.0, and 154.1; ICD-10: C18-C20) were used to identify prevalent or incident CRC either as the first or second diagnosis. Mortality data were extracted from the death registry (follow-up through 31 March 2021). Information on covariates including age, sex, education attainment, ethnicity, family history of bowel cancer, BMI, alcohol intake, and smoking was assessed during the baseline survey. Gender data from UK Biobank was compiled from registry records and self-reporting.^24^

#### Statistical analysis

A multivariable Cox proportional hazards regression model was employed to estimate adjusted hazard ratios (HRs) and 95% confidence intervals (95% CIs) for associations between air pollution exposure and CRC risk and survival. Two levels of statistical adjustments were made: model 1 adjusted for age at recruitment and sex, and model 2 further adjusted for education, Townsend deprivation index (TDI), ethnicity, family history of CRC, BMI, alcohol intake, physical activity and smoking status. Follow-up time was calculated from recruitment until the first recording of the corresponding outcomes (incident CRC diagnosis or all-cause death in patients with CRC), death, date of loss, or end of follow-up, whichever occurred first. The proportional hazards assumption was examined using Schoenfeld residuals and found to be satisfied. The Kaplan–Meier survival analysis was conducted to evaluate the impact of air pollution exposure levels on CRC risk and survival, utilizing the “survminer” R package. Subgroup analyses stratified by sex, smoking status, physical activity and autonomic sites were further conducted to explore potential interactive factors based on prior knowledge. Furthermore, we conducted a sensitivity analysis specifically incorporating patients diagnosed with CRC within 5 years preceding recruitment to mitigate the potential impact of survivorship bias. We also performed a sensitivity analysis after excluding participants with a reported family history of CRC to minimize the potential confounding of familial correlations. The Cox regressions were conducted using the “survival” R package. Wald tests based on Cox proportional hazards models were employed, with a *P*-value of less than 0.05 as statistically significant.

#### Updated meta-analysis of observational studies

The meta-analysis by Pritchett N et al.^11^ reviewed the evidence on outdoor air pollution (mainly in particulate matter) and CRC risk, demonstrated as clinically confirmed CRC diagnosis or CRC-related mortality among the general population. However, this review only included original research with cohort or case–control study design between 1980 and September 2021.^11^ We updated this systematic review to include studies up to January 2023, employing the same methods to study the association between air pollution and CRC endpoints (incidence or CRC-specific mortality). We used PubMed, EMBASE, Cochrane Library, Scopus, and Web of Science for an updated literature search, using keyword describing air pollution exposure (e.g., “outdoor air pollution”, “particulate matter”) and CRC. We employed inverse variance weighted random-effects model to calculate the pooled relative risks (RR) and 95% CIs. Heterogeneity among the studies was quantified using I^2^ test. Meta-analyses were performed with the “metafor” package in R 4.2.0. Details of the search strategies and eligibility criteria were available in the Supplementary Methods.^11^

### Two-sample epigenetic Mendelian randomization

#### mQTL for air pollution-related DNA methylation

We sourced blood DNAm related to PM_2.5_, PM_10_ and NO_2_ exposure from three independent EWAS^15^, ^16^, ^17^ consisting of 2956, 365 and 2410 European participants, respectively. DNAm was measured by the Illumina Infinium HumanMethylation450 (HM450) BeadChip array, and the associations between air pollutants and blood DNAm were adjusted for age, sex, BMI, smoking status, alcohol intake, batch effects, and white blood cell types where applicable.^15^, ^16^, ^17^ A total of 1829, 286 and 4980 CpG sites were associated with PM_2.5_, PM_10_ and NO_2_ exposure, which survived the FDR threshold of *P* < 0.05 at epigenome-wide significance level (Supplementary Table S2).

We then identified CpG site-associated methylation quantitative trait loci (mQTLs) from the Genetics of DNA Methylation Consortium (GoDMC) database, which encompasses genetic and methylation data from over 30,000 individuals.^25^ These mQTLs, serving as genetic proxies for DNA methylation influenced by air pollution, were selected based on the following criteria. We focused on significant cis-mQTLs (*P* < 1 × 10^−8^), located within 1 MB proximity to their respective CpG sites, and exhibiting low linkage disequilibrium (r^2^ < 0.01). F-statistics were also calculated to measure the strength of instruments (F-statistic  <10 was considered as weak instrument). When evaluating the causal effect of genetically predicted DNA methylation at air pollution related CpG sites on CRC, each air pollution-related CpG site was treated as an independent exposure, and its proxy mQTLs in the GoDMC database were used as genetic instruments. These approaches allowed us to effectively proxy the methylation levels of each CpG site associated with exposures to PM_2.5_, PM_10_, and NO_2_.^26^

#### GWAS summary-level data of CRC

GWAS summary statistics for CRC were derived from 12 previously reported GWASs, comprising 20,049 cases and 22,661 controls of European ancestry from the following studies: CCRR1, CCFR2, COIN, CORSA, Croatia, DACHS, FIN, NSCCG OncoArray, SCOT, UK1, VQ58, and Scottish case–control series.^27^ After standard quality control procedures, a total of 16,871 cases and 26,328 controls were included in the meta-GWAS analysis.^27^

#### Two-sample MR

We conducted two-sample MR analysis to elucidate the underlying biology by exploring the causal effects of air pollution-related DNAm on the risk of CRC. For the CpG site with only one mQTL, the Wald ratio was used as the primary analysis.^28^ The false discovery rate (FDR) with the Benjamini-Hochberg method was applied to account for multiple testing, and the association with an FDR-adjusted *P*-value <0.05 was considered statistically significant. The heterogeneity of SNPs' estimates and the pleiotropy of the association was examined by Cochran's Q statistics or MR-Egger intercept test, respectively. Additionally, several sensitivity analyses such as weighted median were conducted to test the consistency of the results.

#### Colocalization of mQTLs and CRC GWAS signals

For CpG sites convincingly associated with the risk of CRC (FDR <0.05), we conducted colocalization analyses to examine whether susceptibility to CRC was driven by the same variants influencing methylation at the CpG sites. The mQTL proxies for methylation at each CpG site and GWAS data for CRC were identical to those used in the MR analysis. Observation of 80% or higher posterior probability of association for both summary effect of the CpG site and single effect of a mQTL were considered as evidence of colocalization. The colocalization analysis was performed using the “coloc” R package.^29^

#### Prospective mQTL-air pollution interaction analyses in UK Biobank

We performed prospective mQTL-air pollution interaction analyses to test whether the epigenetic alterations in mapped gene might modify the effects of air pollution on CRC risk and survival. We retrieved the genotyping data of their mQTLs in the UK Biobank to test whether the associations of air pollution with CRC risk and overall survival, is modified by the genetic polymorphisms of CpG-mapped genes. The interaction analyses were performed using the “CGEN” R package, adjusting for age at recruitment, sex, education, TDI, ethnicity, family history of CRC, BMI, alcohol intake, physical activity, smoking and the first 10 genetic principal components. For the mQTLs significantly interacted with a specific air pollutant or APES, we further conducted stratification analyses to assess the dose–response effects based on their genotypes. All statistical tests were two-tailed and performed using R software 4.2.0. *P*-value <0.05 was considered statistically significant.

### Role of funders

The funding sources had no role in the design of this study and did not have any role in data collection, data analyses, interpretation, writing of report, or decision to submit results.

## Results

### Baseline characteristics of prospective cohort study

After a median of 12.97 years of follow-up, the CRC incidence analysis included 415,887 participants, among which 5767 CRC cases were newly diagnosed. Furthermore, 533 cases of all-cause death were recorded among 2401 patients with CRC. Supplementary Tables S3 and S4 summarized the distribution of baseline characteristics according to the status of incidence and survival.

### Air pollution and risk of CRC incidence

Table 1 presented the results of minimally and fully adjusted HRs for the associations between air pollutants exposure and risk of CRC incidence. No significant associations were observed between individual air pollutant and CRC risk. However, when examining joint exposure to various air pollutants and their effect on CRC incidence, we found a significant association of APES with increased CRC risk in the fully adjusted model (HR = 1.03, 95% CI = 1.01–1.06). The results remained consistent after excluding participants with a reported family history of CRC (Supplementary Table S5). Adjusted Kaplan–Meier survival analysis (Supplementary Figure S2) showed suggestive differences in CRC incidence across diverse levels of PM_10_ (log-rank *P* = 0.007) and NO_x_ (log-rank *P* = 0.027).

**Table 1:** Associations between individual and combined exposure to air pollution and risk of CRC incidence in UK Biobank.

Pollution	Cases	Model 1		Model 2	
		HR (95% CI)	*P* value	HR (95% CI)	*P* value
**PM**_**2.5**_**(μg/m3)**					
Q1	1499	1.00 (ref)		1.00 (ref)	
Q2	1436	0.97 (0.90, 1.04)	0.386	0.98 (0.91, 1.05)	0.565
Q3	1469	1.04 (0.97, 1.12)	0.300	1.05 (0.98, 1.13)	0.192
Q4	1363	1.00 (0.93, 1.08)	0.939	1.01 (0.94, 1.09)	0.776
P for trend			0.595		0.483
Per 5-μg/m3∗day		1.08 (0.95, 1.22)	0.239	1.09 (0.95, 1.24)	0.211
**PM**_**10**_**(μg/m3)**					
Q1	1505	1.00 (ref)		1.00 (ref)	
Q2	1469	1.00 (0.93, 1.07)	0.894	1.00 (0.93, 1.08)	0.997
Q3	1473	1.03 (0.96, 1.10)	0.463	1.03 (0.96, 1.11)	0.376
Q4	1320	0.97 (0.90, 1.05)	0.480	1.00 (0.92, 1.08)	0.920
P for trend			0.616		0.915
Per 5-μg/m3∗day		0.98 (0.91, 1.04)	0.478	1.00 (0.93, 1.08)	0.933
**NO**_**2**_**(μg/m3)**					
Q1	1494	1.00 (ref)		1.00 (ref)	
Q2	1481	1.00 (0.93, 1.08)	0.961	1.01 (0.94, 1.08)	0.806
Q3	1439	1.01 (0.94, 1.08)	0.882	1.01 (0.94, 1.09)	0.696
Q4	1353	1.00 (0.93, 1.08)	0.900	1.03 (0.95, 1.12)	0.451
P for trend			0.885		0.447
Per 5 μg/m3∗day		1.00 (0.99, 1.01)	0.974	1.01 (0.99, 1.02)	0.385
**NO**_**x**_**(μg/m3)**					
Q1	1534	1.00 (ref)		1.00 (ref)	
Q2	1453	0.97 (0.90, 1.04)	0.352	0.97 (0.9, 1.04)	0.442
Q3	1386	0.95 (0.89, 1.03)	0.197	0.96 (0.9, 1.04)	0.339
Q4	1394	1.01 (0.94, 1.09)	0.785	1.02 (0.95, 1.11)	0.572
P for trend			0.833		0.620
Per 5-μg/m3∗day		1.00 (0.99, 1.01)	0.730	1.00 (0.99, 1.01)	0.489
**Combined air pollution exposure score** (per SD increment)	5767	1.04 (1.01, 1.07)	0.008	1.03 (1.01, 1.06)	0.016

Model 1 adjusted for age at recruitment and sex.

Model 2 adjusted for age at recruitment, sex, education, Townsend deprivation index (TDI), ethnicity, family history of CRC, BMI, alcohol intake, physical activity and smoking.

Further stratification analyses by sex, smoking behaviors and physical activity revealed significant associations between APES and CRC risk in men (HR = 1.01, 95% CI = 1.00–1.01), ever or current smokers (HR = 1.05, 95% CI = 1.01–1.08), and in those with insufficient physical activity (HR = 1.05, 95% CI = 1.01–1.08) (P_interaction_ > 0.05, details in Supplementary Tables S6–S9). Besides, the adverse effect of APES was significant for colon cancer risk (HR = 1.04, 95% CI = 1.00–1.08), but not for rectal cancer risk.

### Air pollution and risk of all-cause mortality in CRC

As shown in fully adjusted models from Table 2, compared to those exposed to the lowest quartile of air pollutants, exposure to the highest quartile of air pollutants was generally associated with an increased risk of all-cause mortality among CRC survivors (HR = 1.43, 95% CI = 1.10–1.87, P_trend_ = 0.011 for PM_2.5_; HR = 1.52, 95% CI = 1.16–2.00, P_trend_ = 0.005 for NO_2_, HR = 1.43, 95% CI = 1.10–1.86, P_trend_ = 0.008 for NO_x_), although not all risk estimates were statistically significant. Per 5 μg/m^3^ increment in PM_10_, the risk of all-cause mortality was 28% higher (HR = 1.28, 95% CI = 1.02–1.62) among individuals with CRC. Moreover, the joint exposure to various air pollutants was associated with an elevated risk of mortality in CRC survivors (HR = 1.13, 95% CI = 1.03–1.23). Similar results were found by the adjusted Kaplan–Meier curves in Supplementary Figure S3. These analyses indicated that increased exposure to specific air pollutants, particularly within the higher quartiles, was associated with decreased survival probability in patients with CRC [(log-rank *P* = 0.004), NO_2_ (log-rank *P* = 0.001), NO_x_ (log-rank *P* = 0.006), and the APES (log-rank *P* = 0.008)]. The results remained consistent both for patients diagnosed with CRC within the five-year period prior to their recruitment (Supplementary Table S10), and after excluding CRC cases with a reported family history of CRC (Supplementary Table S11).

**Table 2:** Associations between individual and combined exposure to air pollution and risk of all-cause mortality among CRC survivors in UK Biobank.

Pollution	Cases	Model 1		Model 2	
		HR (95% CI)	*P* value	HR (95% CI)	*P* value
**PM**_**2.5**_**(μg/m3)**					
Q1	104	1.00 (ref)		1.00 (ref)	
Q2	140	1.40 (1.08, 1.80)	0.010	1.34 (1.04, 1.73)	0.024
Q3	136	1.42 (1.10, 1.84)	0.007	1.35 (1.04, 1.76)	0.023
Q4	153	1.56 (1.21, 2.00)	0.001	1.43 (1.10, 1.87)	0.008
P for trend			0.001		0.011
Per 5-μg/m3∗day		1.77 (1.23, 2.55)	0.002	1.54 (1.04, 2.29)	0.031
**PM**_**10**_**(μg/m3)**					
Q1	124	1.00 (ref)		1.00 (ref)	
Q2	129	1.05 (0.82, 1.34)	0.712	1.02 (0.79, 1.3)	0.903
Q3	134	1.10 (0.86, 1.41)	0.427	1.04 (0.81, 1.33)	0.758
Q4	146	1.27 (1.00, 1.61)	0.051	1.19 (0.93, 1.53)	0.172
P for trend			0.042		0.156
Per 5-μg/m3∗day		1.34 (1.08, 1.67)	0.008	1.28 (1.02, 1.62)	0.037
**NO**_**2**_**(μg/m3)**					
Q1	99	1.00 (ref)		1.00 (ref)	
Q2	139	1.50 (1.16, 1.94)	0.002	1.45 (1.12, 1.88)	0.005
Q3	149	1.61 (1.25, 2.08)	0.000	1.54 (1.19, 2.00)	0.001
Q4	146	1.62 (1.25, 2.09)	0.000	1.52 (1.16, 2.00)	0.003
P for trend			0.000		0.005
Per 5 μg/m3∗day		1.07 (1.03, 1.12)	0.002	1.06 (1.01, 1.12)	0.016
**NO**_**x**_**(μg/m3)**					
Q1	106	1.00 (ref)		1.00 (ref)	
Q2	131	1.28 (0.99, 1.66)	0.056	1.25 (0.97, 1.62)	0.089
Q3	142	1.42 (1.11, 1.83)	0.006	1.35 (1.04, 1.75)	0.023
Q4	154	1.53 (1.19, 1.96)	0.001	1.43 (1.10, 1.86)	0.008
P for trend			0.001		0.008
Per 5-μg/m3∗day		1.04 (1.01, 1.07)	0.003	1.03 (1.00, 1.06)	0.027
**Combined air pollution exposure score** (per SD increment)	533	1.14 (1.06, 1.24)	0.001	1.13 (1.03, 1.23)	0.011

Model 1 adjusted for age at recruitment and sex.

Model 2 adjusted for age at recruitment, sex, education, TDI, ethnicity, family history of CRC, BMI, alcohol intake, physical activity and smoking.

When stratified by sex, smoking behaviours, physical activity and tumour subsite (Supplementary Tables S12–S15), the associations with PM_2.5_, NO_2_, NO_x_ and APES were statistically significant in men, individuals with insufficient physical activity, and survivors of colon cancer, and the associations with oxynitride and APES remained stable in current or ever smokers.

### Updated meta-analysis

Two studies published between September 2021 and January 2023 were included in the updated meta-analysis following full-text screening. More details regarding the process of screening are shown in Supplementary Figure S4. These two studies, conducted in South America and Asia, both reported on the association between PM and risk of CRC-specific mortality among general population. Along with four earlier studies, all included studies were retrospective or prospective cohort analyses estimating associations with PM_2.5_, hence we only summarized PM_2.5_ associations (Details of included study were shown in Supplementary Table S16).

**Fig. 2:**
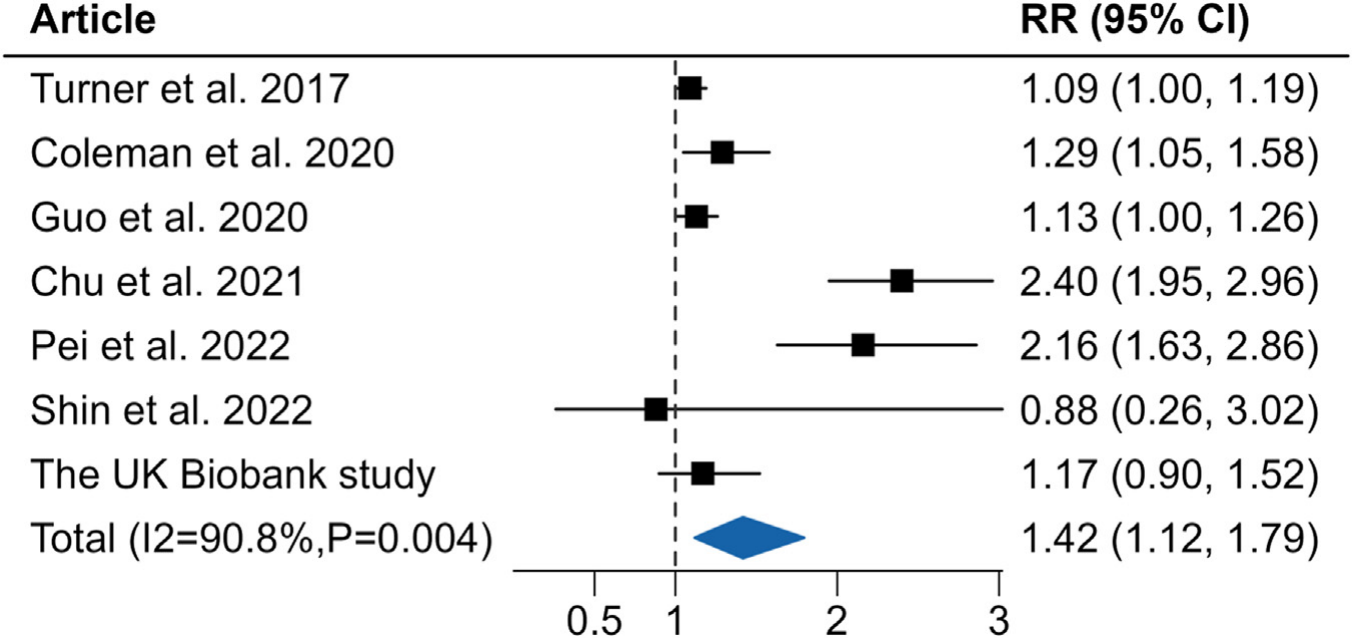
**Meta-analysis of included epidemiologic studies.** Reported effect estimates (95% CI) from individual studies and overall pooled estimate (including the *P* value) from random-effects (RE) model for PM_2.5_ exposure (10 μg/m^3^ increase) and CRC.

Fig. 2 presented the pooled HR estimates per 10 μg/m^3^ increment in PM_2.5_ (RR = 1.47, 95% CI = 1.12–1.92; I^2^ = 92.3%) and CRC risk, including four previous studies incorporated in the research by Pritchett N et al. When our current results based on UK Biobank data were included, the association between per 10 μg/m^3^ increase in PM_2.5_ and the risk of CRC remained stable (RR = 1.42, 95% CI = 1.12–1.79; I^2^ = 90.8%) (Fig. 2). Since outdoor air pollution varied significantly regionally in both overall burden and its constituency, the considerable heterogeneity observed might be largely attributable to geographic and ancestry heterogeneity in included studies (i.e., United States, South America, Europe, and China) of our meta-analyses.

### Causal effect of air pollution-related DNAm on CRC risk

Initially, 1829, 286 and 4980 CpG sites were identified to be associated with PM_2.5_, PM_10_ and NO_2_ exposure from studies with participants of European ancestry^15^, ^16^, ^17^ (Supplementary Table S2). After applying rigorous selection criteria to ensure the robustness of our genetic instruments, we found 580 CpG sites associated with PM_2.5_ exposure and their 1022 corresponding mQTLs, 49 CpG sites for PM_10_ exposure and their 123 corresponding mQTLs, and 3006 CpG sites for NO_2_ exposure and their 10,676 corresponding mQTLs (Supplementary Tables S17–S19). Table 3 presented the MR estimates of genetically predicted blood DNAm at each CpG site on CRC incidence risk. Genetically predicted methylation at PM_2.5_-related CpG site cg13835894 [*TMBIM1/PNKD*] and cg16235962 [*CXCR5*] were associated with elevated CRC risk, with MR estimates being 1.19 (95% CI = 1.12–1.27) and 1.54 (95% CI = 1.27–1.88), respectively. For NO_2_, the Wald ratio approach suggested that genetically predicted methylation at CpG site cg16947394 [*TMEM110*] was linked to increased risk of CRC (OR = 1.57, 95% CI = 1.30–1.89). No apparent heterogeneity was detected for PM_2.5_-related cg13835894 (Cochrane's Q value = 0.012).

**Table 3:** Two-sample Mendelian Randomization estimates of air pollution-related methylation on CRC risk.

Exposure	CpG site	CpG.Chr	CpG.Pos	Nearest gene	Method	SNP	OR (95% CI)	*P* value	FDR
PM_2.5_	cg13835894	2	219,148,914	*TMBIM1/PNKD*	IVW	rs1870123, rs876961	1.19 (1.12, 1.27)	5.04E-09	4.43E-06
	cg16235962	11	118,754,507	*CXCR5*	Wald ratio	rs497916	1.54 (1.27, 1.88)	1.15E-05	0.005
NO_2_	cg16947394	3	52,930,628	*TMEM110*	Wald ratio	rs11708390	1.57 (1.30, 1.89)	1.88E-06	0.004

Chr, chromosome; Pos, position; OR, odds ratio, CRC risk per SD change in DNA methylation at air pollution related CpG sites in blood; 95% CI, 95% confidence interval; IVW, inverse variance weighted.

Among the three CpG sites significantly associated with CRC risk, methylation at cg16235962, mapped to gene *CXCR5*, had a 99.7% posterior probability of sharing a causal variant (rs497916) with CRC GWAS signals (Supplementary Figure S5). To investigate whether the mQTLs of three air pollution-related CpG sites would influence the expression of their mapped gene in colon tissue, we queried the GTEx Portal and found that the mQTL rs1870123 of cg13835894 is an eQTL of the TMBIM1 gene (m-value = 1.00) (Supplementary Figures S6–S8).

### Prospective mQTL-air pollution interaction effects on CRC risk and survival

Table 4 displayed the prospective mQTL-air pollution interaction effect estimates on CRC risk and survival. Three mQTLs had evidence for interaction with APES on CRC risk or survival, while only rs876961 of cg13835894 [*TMBIM1/PNKD*] significantly interacted with PM_2.5_ exposure on CRC survival.

**Table 4:** Gene-air pollution interaction estimates for CRC risk and survival in the UK Biobank.

Variables	Risk of CRC incidence				Risk of all-cause mortality among CRC survivors			
	Model 1		Model 2		Model 1		Model 2	
	HR (95% CI)	*P* value	HR (95% CI)	*P* value	HR (95% CI)	*P* value	HR (95% CI)	*P* value
**mQTLs of air pollution related methylation**								
rs1870123	1.07 (1.03, 1.11)	0.001	1.07 (1.02, 1.11)	0.002	1.09 (0.94, 1.26)	0.278	1.08 (0.93, 1.26)	0.309
rs876961	1.02 (0.98, 1.06)	0.338	1.02 (0.98, 1.06)	0.357	0.98 (0.85, 1.13)	0.811	1.00 (0.86, 1.15)	0.965
rs497916	1.00 (0.96, 1.05)	0.839	1.01 (0.97, 1.06)	0.565	1.19 (1.02, 1.40)	0.032	1.20 (1.02, 1.42)	0.024
rs11708390	1.03 (0.98, 1.09)	0.252	1.03 (0.97, 1.09)	0.332	0.84 (0.68, 1.03)	0.094	0.82 (0.66, 1.01)	0.057
**Gene-individual air pollutant interaction effect**								
rs1870123 × PM_2.5_ (5-μg/m3∗day)	1.11 (0.93, 1.32)	0.244	1.16 (0.95, 1.40)	0.138	1.49 (1.09, 2.02)	0.011	1.35 (0.98, 1.88)	0.067
rs876961 × PM_2.5_ (5-μg/m3∗day)	1.13 (0.89, 1.42)	0.307	1.17 (0.91, 1.51)	0.208	1.86 (1.21, 2.85)	0.005	1.72 (1.10, 2.69)	0.018
rs497916 × PM_2.5_ (5-μg/m3∗day)	1.18 (0.87, 1.59)	0.286	1.14 (0.83, 1.57)	0.422	1.45 (1.08, 1.94)	0.013	1.32 (0.97, 1.80)	0.079
rs11708390 × NO_2_ (5-μg/m3∗day)	1.00 (0.99, 1.02)	0.774	1.00 (0.98, 1.02)	0.943	1.04 (1.01, 1.07)	0.015	1.03 (1.00, 1.07)	0.062
**Gene-APES interaction effect**								
rs1870123 × APES (per SD)	1.03 (1.01, 1.05)	0.005	1.03 (1.01, 1.05)	0.008	1.09 (1.02, 1.17)	0.010	1.08 (1.00, 1.16)	0.043
rs876961 × APES (per SD)	1.02 (0.99, 1.05)	0.129	1.02 (0.99, 1.05)	0.169	1.10 (1.00, 1.20)	0.047	1.08 (0.98, 1.19)	0.116
rs497916 × APES (per SD)	1.05 (1.01, 1.09)	0.006	1.05 (1.01, 1.09)	0.006	1.08 (1.01, 1.15)	0.022	1.06 (0.99, 1.14)	0.092
rs11708390 × APES (per SD)	1.02 (1.00, 1.03)	0.024	1.02 (1.00, 1.04)	0.024	1.08 (1.02, 1.14)	0.005	1.07 (1.01, 1.14)	0.029

Model 1 adjusted for age at recruitment and sex.

Model 2 adjusted for age at recruitment, sex, education, TDI, ethnicity, family history of CRC, BMI, alcohol intake, physical activity, smoking and the first 10 genetic principal components.

Stratification analyses based on the genotypes of these four mQTLs were shown in Supplementary Tables S20–S21. A per SD increase in APES among participants with rs1870123 AA, rs497916 TC or rs11708390 CC genotypes was associated with both increased CRC risk and poorer survival, with relative risks of 1.07 (95% CI = 1.02–1.12), 1.19 (95% CI = 1.04–1.36) and 1.08 (95% CI = 1.03–1.13) for incidence and 1.34 (95% CI = 1.16–1.55), 1.04 (95% CI = 1.00–1.17) and 1.11 (95% CI = 1.00–1.24) for survival, respectively. For individuals carrying one risk allele of CRC, a per 5 μg/m^3^ increase in PM_2.5_ conferred 112% higher risk of CRC incidence among individuals with rs876961 GA genotypes.

## Discussion

In this study, we comprehensively examined the associations of long-term air pollution exposure, air pollution related DNAm, and the risk and survival of CRC. We utilized an integrated approach that encompassed a prospective cohort study design, an updated meta-analysis and epigenetic MR analysis. Genetic colocalization analyses and gene–environment interaction analyses were also performed to unveil the carcinogenesis or prognosis effects of air pollution on CRC in the context of epigenetic modifications. Our findings suggest a significant association between air pollution and both the CRC incidence and survival. Notably, the methylation occurring within protein-coding genes *TMBIM1/PNKD*, *CXCR5* and *TMEM110* may partially mediate the pathogenic effects of air pollution on CRC.

We created an APES by weighted summations to evaluate joint exposure of various air pollutants and detected a robust and replicable association of APES with an elevated CRC risk as well as poorer survival, suggesting a detrimental overall effect of air pollution exposure on CRC carcinogenesis and prognosis. The subsequent meta-analysis involving data from the UK Biobank and six other multi-ancestry studies consistently support the positive association between PM2.5 exposure and increase CRC risk. However, evidence regarding other air pollutants remained relatively limited and substantial heterogeneity existed, possibly attributed to the extensive geographic diversity and ancestry variations in included studies. For overall survival in CRC survivors, we found that exposure to various air pollutants, individually or jointly as an APES, was associated with poorer CRC survival in a dose–response pattern, a phenomenon not previously investigated by epidemiological studies.^30^ Also, though none of the differences among stratification subgroups reached statistical significance, the adverse carcinogenic or prognostic effects of air pollution appeared to be predominantly among men, ever smokers, and those with insufficient physical activity, suggesting the potential modifying effects of lifestyle factors. In line with our findings, studies linking air pollution with diabetes mellitus,^31^ chronic obstructed pulmonary disease^32^ and cardiometabolic multimorbidity^33^ have consistently shown that adherence to a healthy lifestyle may mitigate the detrimental health effects of air pollution.

Previous studies have suggested that air pollution may contribute to the development of CRC via the systemic inflammatory pathway, which is linked to blood proinflammatory activity and significantly elevated mRNA and protein levels of interferon-γ and interleukin production.^34^ Our exploration of air pollution-induced DNAm offers a new perspective for understanding the etiological effects of air pollutants on CRC in the context of epigenetic modifications. When exploring the effects of genetically predicted air pollution-related methylation on CRC risk, cg13835894 [*TMBIM1/PNKD*], cg16235962 [*CXCR5*] and cg16947394 [*TMEM110*] were identified to elevate CRC risk through epigenetic modification. Moreover, our gene–environment interaction analyses revealed interaction effects with APES at mQTLs of all three aforementioned CpG sites on both CRC risk and survival, and with PM_2.5_ exposure at rs876961 of cg13835894 [*TMBIM1/PNKD*] on CRC survival.

On a population level, prolonged PM_2.5_ exposure has been associated with elevated C-reactive protein levels and an activated systemic inflammatory state.^35^ It has been proposed that PM_2.5_ exposure may exacerbate alterations in intestinal microbial compositions, leading to higher permeability, impaired gut barrier function, inflammatory cell infiltration, and systemic inflammation.^36^ The PM_2.5_-related CpG site cg16235962 was annotated to the gene *CXCR5*. This gene encodes the C-X-C motif chemokine receptor 5, which serves as the G-protein-coupled receptor of CXCL13, an important inflammatory factor in the microenvironment.^37^ The CXCL13-CXCR5 signaling axis is expressed mainly in both T and B cells, where it regulates lymphocyte migration and promotes inflammation.^37^ Consistent with the proinflammatory pathological effects of air pollutants, this axis is implicated in chronic inflammation, infectious diseases, autoimmune diseases, and tumors.^37^ It has been proposed to promote the growth and invasion of colon cancer cells, probably via the PI3K/AKT pathway,^38^ and has been reported to be associated with poor prognosis in advanced colon cancer, suggesting the potential utility of CXC family chemokines as prognostic or predictive biomarkers and possible drug targets in CRC.^39^

Another PM_2.5_-related CpG site cg16947394 is linked to *TMBIM1*, encoding the transmembrane BAX inhibitor motif-containing 1 protein.^40^ The variant rs992157, located in the intron of *PNKD* and *TMBIM1*, was found to be significantly associated with the susceptibility and progression of CRC in both European and Chinese populations, possibly via the up-regulation of *TMBIM1*.^41^^,^^42^ Similarly, disrupted *PNKD* function may reduce glutathione levels, increasing oxidative stress and inflammation.^43^ *TMBIM1* has been reported to regulate Fas ligand levels, affect apoptosis and inflammation.^44^ Thus, *PNKD* and *TMBIM1* may indirectly influence inflammation regulation, a key process in inflammatory bowel disease and CRC development, suggesting their potential impact on CRC via inflammation pathways. Furthermore, the mQTL rs1870123 of CpG site cg13835894 are strong eQTLs of the *TMBIM1* gene in colon tissue, indicating that alterations in methylation at cg13835894 could potentially influence CRC risk through modulation of *TMBIM1* gene expression. Our methylation MR and gene–environment interaction analyses offer new insights into the role of the *TMBIM1* gene in CRC development from the perspectives of air pollution and DNAm.

Additionally, the transmembrane protein 110 encoded by *TMEM110* was considered to regulate the long-term maintenance of endoplasmic reticulum-plasma membrane junctions and the short-term physiological remodeling of the junctions during store-dependent calcium signalling.^45^ A proteome profiling study of benign and malignant pulmonary nodules identified *TMEM110* as a candidate biomarker for non-invasive molecular diagnosis of lung cancer due to its significant nodule-specific abundance.^46^ However, there was no relevant evidence of *TMEM110* in the context of CRC. Our study provided evidence for its interaction with NO_2_, joint air pollution exposure and their pathogenic effects on CRC utilizing mQTLs, for which future functional experiment are crucial to validate our findings and to further elucidate the role of *TMEM110* in CRC development.

Our findings underscored the significance of a comprehensive evaluation of multiple air pollutants and the implementation of health interventions targeting various modifiable environmental factors (e.g., air pollution, smoking, physical activity) in strengthening both primary and secondary prevention for CRC. The profound influence of epigenetic alterations on the complex pathogenesis of CRC was also emphasized. However, the underlying mechanisms by which these alterations contribute to CRC development still necessitate further exploration.

The strengths of the study include utilization of a nationally representative, well-characterized, large-scale cohort study population and an integrative approach combining a prospective cohort study, an updated meta-analysis, and an epigenetic MR analysis that enabled causal inferences linking air pollution, DNAm and CRC. The present study investigate the individual or joint effects of various air pollution on CRC risk and survival, with a particular focus on the epigenetic modification and the gene–environment interaction insights. However, some limitations need to be acknowledged. First, the CpG sites associated with air pollution were obtained from cross-sectional EWASs, thereby limited the possibility to investigate the life course or dynamic change of air pollution exposure on DNAm. Second, methylation data were derived from blood samples. Although blood DNAm is the most extensively investigated epigenetic makers,^47^ the methylation signature may differ across peripheral blood and specific tissues. Third, our study was unable to directly test for the mediation effects linking air pollution to CRC due to absence of individual-level DNA methylation data. Future studies with detailed methylation profiles to directly assess the potential mediating effects between air pollution and CRC are warranted. Moreover, it is imperative for further studies to incorporate individual-level methylation profiling of CpG sites, alongside gene expression data, to elucidate the intricate epigenetic mechanisms influencing gene function and carcinogenesis. Fourth, even though we adjusted for several potential confounding factors (e.g., sex, education level, family history of CRC), we lacked information on CRC stage at diagnosis, a critical determinant of survival for CRC.^48^ Lastly, our prospective analysis is limited to UK Biobank participants, necessitating future research with larger and more diverse populations, given the significant regional variations in outdoor air pollution in terms of both total burden and composition.^11^ The differential methylation patterns among ethnic subgroups warrant further investigations.^49^

### Conclusion

In conclusion, our study provided comprehensive evidence supporting a detrimental impact of ambient air pollution on CRC risk and survival, highlighting the powerful modifying effects of epigenomic factors. The pathogenic effect of air pollution may be partly attributed to the epigenetic alterations of TMBIM1/PNKD, CXCR5, and TMEM110, where mQTL polymorphisms could modify the effects of air pollution on CRC risk and survival. This research offered new insights from epigenetic and gene–air interaction perspectives to understand the complex pathogenesis of CRC, necessitating future research with tissue-specific datasets to elucidate the underlying mechanisms.

## Contributors

X.L. and E.T. conceptualized the project. F.J., W.C., and Y.Z. performed the literature review, the data analyses and wrote the first draft. J.Z. and J.S. accessed and verified the data. The manuscript was revised and edited by X.L., E.T., S.Y., S.C.L, M.G.D. and F.J., J.Z., J.S., W.C., Y.Z., S.Z., M.T., P.J.L., C.D., and R.S.H. interpreted data, reviewed the paper, and made critical revision of the manuscript for important intellectual content. X.L., E.T., and M.G.D. are the study guarantor. All authors read and approved the final version of the manuscript.

## Data sharing statement

The results of this study are included in this article and the Supporting files. The UK Biobank is an open access resource and researchers required approval from the UK Biobank (www.ukbiobank.ac.uk/). Genetic data was derived from the large genome-wide association study (GWAS), Genetics of DNAm Consortium (GoDMC) and genome-wide DNAm analyses, which can be required from the published articles.

## Declaration of interests

We declare no competing interests.
