# Supplementary material for: Impact of ambient air pollution on colorectal cancer risk and survival: insights from a prospective cohort and epigenetic Mendelian randomization study
Source: eBioMedicine. 2024 Apr 16;103:105126. doi: 10.1016/j.ebiom.2024.105126 (PMC11035091; doi:10.1016/j.ebiom.2024.105126)
Supplement: Supplementary Figures S1–S8 [file mmc1.docx]

**Supplementary Material**

**Supplementary Methods**

**Supplementary Tables**

**Supplementary Table 1.** Assessment of Air pollution in UK Biobank

**Supplementary Table 2.** Air pollution related EWAS study and CpG sites

**Supplementary Table 3.** Baseline characteristics of study participants in CRC incidence analysis.

**Supplementary Table 4.** Baseline characteristics of study participants in CRC survival analysis.

**Supplementary Table 5.** Associations between individual and combined exposure to air pollution and risk of CRC incidence in UK Biobank, excluding participants with a reported family history of CRC.

**Supplementary Table 6.** Associations between individual and combined exposure to air pollution and risk of CRC incidence stratified by gender in UK Biobank.

**Supplementary Table 7.** Associations between individual and combined exposure to air pollution and risk of CRC incidence stratified by smoking status in UK Biobank.

**Supplementary Table 8.** Associations between individual and combined exposure to air pollution and risk of CRC incidence stratified by physical activity in UK Biobank.

**Supplementary Table 9.** Associations between individual and combined exposure to air pollution and risk of CRC incidence stratified by anatomic sites in UK Biobank.

**Supplementary Table 10.** Associations between air pollution and risk of all-cause mortality from CRC diagnosed within 5 years.

**Supplementary Table 11.** Associations between individual and combined exposure to air pollution and risk of all-cause mortality among CRC survivors in UK Biobank , excluding participants with a reported family history of CRC.

**Supplementary Table 12.** Associations between individual and combined exposure to air pollution and risk of all-cause mortality in CRC survivors stratified by gender in UK Biobank.

**Supplementary Table 13.** Associations between individual and combined exposure to air pollution and risk of all-cause mortality in CRC survivors stratified by smoking status in UK Biobank.

**Supplementary Table 14.** Associations between individual and combined exposure to air pollution and risk of all-cause mortality in CRC survivors stratified by physical activity in UK Biobank.

**Supplementary Table 15.** Associations between individual and combined exposure to air pollution and risk of all-cause mortality in CRC survivors stratified by anatomic sites in UK Biobank.

**Supplementary Table 16.** Articles and reported effect estimates included in the meta-analysis of PM exposure and CRC.

**Supplementary Table 17.** PM2.5-related CpG sites and mQTLs from GoDMC database.

**Supplementary Table 18.** PM10-related CpG sites and mQTLs from GoDMC database.

**Supplementary Table 19.** NO2-related CpG sites and mQTLs from GoDMC database.

**Supplementary Table 20.** Dose-response effect of combined air pollution on the risk of CRC incidence stratified by the genotype of rs1870123, rs876961,rs497916 and rs11708390 in the UK Biobank.

**Supplementary Table 21.** Dose-response effect of individual or combined air pollution on the CRC survival stratified by the genotype of rs1870123, rs876961,rs497916 and rs11708390 in the UK Biobank.

**Supplementary Figures**

**Supplementary Figure 1.** Flowchart of population selection in the prospective cohort study.

**Supplementary Figure 2.** Kaplan-Meier analysis for long-term impact of air pollution on CRC incidence in the UK Biobank.

**Supplementary Figure 3.** Kaplan-Meier analysis for long-term impact of air pollution on CRC survival in the UK Biobank.

**Supplementary Figure 4.** PRISMA flow diagram showing the literature search and screening process for studies relevant to PM exposure and CRC outcomes (published between September 2021 and January 2023).

**Supplementary Figure 5.** Regional plot for colocalization of methylation at cg16235962 [CXCR5] and CRC susceptibility.

**Supplementary Figure 6.** Single-tissue eQTL plot of rs1870123 (cg13835894, TMBIM1).

**Supplementary Figure 7.** Single-tissue eQTL plot of rs1870123 (cg13835894, PNKD).

**Supplementary Figure 8.** Single-tissue eQTL plot of rs11708390 (cg16947394, TMEM110).

**Supplementary Methods**

**Updated meta-analysis of observational studies**

***literature search***

This study was conducted according to PRISMA (The Preferred Reporting Items for Systematic Reviews and Meta-Analyses) guidelines. Based on the existing research termed *“Exposure to Outdoor Particulate Matter Particulate pollutants and Risk of Gastrointestinal Cancers in Adults: A Systematic Review and Meta-Analysis of Epidemiologic Evidence”^[1]^*, We performed an updated search of all the English literature on the topic of particulate pollutants and CRC published during September 2021 to January 2023. The updated search was conducted by replication method in the following databases: Embase, PubMed, Scopus, Web of Science and Cochrane library, the detailed search strategies can be found elsewhere. EndNote X20 was used to collect, manage, and identify duplicate literature.

***Inclusion and exclusion***

Studies were included if they met the following criteria: (i) examined the relationship between particulate pollutants and CRC incidence or mortality; (ii) reported odds ratio (OR), relative risk (RR) or hazard ratio (HR) for every increase of unit (μg/m^3^) in the concentration of particulate pollution; (iii) reported standard errors or confidence intervals (CIs) of the risk estimates (or providing sufficient data to calculate them). Studies were excluded if they meet the following criteria: (i) not case-control study or cohort study; (ii) did not report the independent effect of particulate pollution on CRC risk; (iii) full text was not available; (iv) reported the risk estimates for different levels of PM exposure only (e.g., high vs low exposure); (v) when more than one study was published for the same cohort, only the most recent and comprehensive one was included and the others were excluded.

First, two authors independently did the initial screening of the title and abstract following the criteria above, which mainly excluded the articles have wrong expose and outcome. Next, the full-text screening was done independently using following stricter criteria: an association between PM and CRC end point (incidence or mortality) in adults (≥18 years of age) was evaluated with a cohort or case-control study design. Studies with wrong type or unavailable effect estimates for PM with concurrent standard errors or CI were excluded in this step. The flow diagram of literature search and screening process were shown in **Supplementary Figure 1**.

***Data extraction and Statistical analyses***

Two authors independently extracted data needed from every included article using Excel. Any disagreement was resolved by discussion or in consultation with a third author. The descriptive characteristics extracted from each article were: PMID, author, year published, study design, study population, exposure window and unit, outcome, and the reported measure of association. We extracted fully adjusted regression estimates and 95% CIs for use in meta-analysis.

Meta-analyses were performed using the “metafor” package for R. We chose random effects model to pool the data according to the test result. Heterogeneity among the studies was quantified using I^2^ test. Forrest plots were used to show pooled estimates and 95% CI.

[1] PRITCHETT N, SPANGLER E C, GRAY G M, et al. Exposure to Outdoor Particulate Matter Air Pollution and Risk of Gastrointestinal Cancers in Adults: A Systematic Review and Meta-Analysis of Epidemiologic Evidence [J]. Environ Health Perspect, 2022, 130(3): 36001.

**Supplementary Tables**

**Supplementary Table 1. Assessment of Air pollution in UK Biobank**

| **Exposure** | **Assessment** | **UKB ID** |
| --- | --- | --- |
| NO_2_ | Mean value of NO_2_ measured in 4 years | 24016, 24017, 24018, 24003 |
| NO_X_ | Sigle measurement in 2010 | 24004 |
| PM_10_ | Mean value of NO_2_ measured in 2 years | 24019, 24005 |
| PM_2.5_ | Sigle measurement in 2010 | 24006 |

**Supplementary Table 2. Air pollution related EWAS study and CpG sites**

|  | **Exposures** | **EWAS study** | **Sample size** | **CpG sites** |
| --- | --- | --- | --- | --- |
| **Particulate matter** | PM_2.5_ | Panni T, Mehta AJ, Schwartz JD, et al. Genome-Wide Analysis of DNA Methylation and Fine Particulate Matter Air Pollution in Three Study Populations: KORA F3, KORA F4, and the Normative Aging Study. Environ Health Perspect. 2016;124(7):983-990. doi:10.1289/ehp.1509966 | 3 cohorts (N=2956) | 1829 (FDR<0.05) |
|  | PM_10_ | Isaevska E, Fiano V, Asta F, et al. Prenatal exposure to PM_10_ and changes in DNA methylation and telomere length in cord blood. Environ Res. 2022;209:112717. doi:10.1016/j.envres.2022.112717 | 1 cohort (N=365) | 285 (FDR<0.05) |
| **Oxynitride** | NO_2_ | de F C Lichtenfels AJ, van der Plaat DA, de Jong K, et al. Long-term Air Pollution Exposure, Genome-wide DNA Methylation and Lung Function in the LifeLines Cohort Study. Environ Health Perspect. 2018;126(2):027004. Published 2018 Feb 6. doi:10.1289/EHP2045 | 3 cohorts (N=2410) | 4980 (FDR<0.05) |

EWAS, epigenome-wide association studies.

**Supplementary Table 3. Baseline characteristics of study participants in CRC incidence analysis.**

| **Characteristics** | **Non-cases** | **CRC cases** |
| --- | --- | --- |
|  | (n=422865) | (n=5767) |
| **Recruitment age (mean (SD))** | 56.21 (8.11) | 60.51 (6.67) |
| **Gender (%)** |  |  |
| Female | 228898 (54.1) | 2534 (43.9) |
| Male | 193967 (45.9) | 3233 (56.1) |
| **Education (%)** |  |  |
| College | 133913 (31.7) | 1646 (28.5) |
| Miss | 8808 (2.1) | 156 (2.7) |
| Non-College | 280144 (66.2) | 3965 (68.8) |
| **Townsend deprivation index (%)** | | |
| Low | 211557 (50.0) | 3023 (52.4) |
| High | 211308 (50.0) | 2744 (47.6) |
| **Family history of bowel cancer (%)** | | |
| No | 293479 (69.4) | 3694 (64.1) |
| Yes | 28257 (6.7) | 583 (10.1) |
| Missing | 101129 (23.9) | 1490 (25.8) |
| **Ethnicity (%)** |  |  |
| Non-White | 24823 (5.9) | 187 (3.3) |
| White | 395608 (94.1) | 5546 (96.7) |
| **BMI (%)** |  |  |
| Abnormal | 283627 (67.1) | 4194 (72.7) |
| Normal | 136586 (32.3) | 1538 (26.7) |
| Missing | 2652 (0.6) | 35 (0.6) |
| **Smoking status (%)** |  |  |
| Current / ever smoking | 188735 (44.6) | 3082 (53.4) |
| Never smoking | 231617 (54.8) | 2657 (46.1) |
| Missing | 2513 (0.6) | 28 (0.5) |
| **Physical activity (%)** |  |  |
| Insufficient | 298230 (70.5) | 4118 (71.4) |
| Sufficient | 96291 (22.8) | 1232 (21.4) |
| Missing | 28344 (6.7) | 417 (7.2) |
| **Alcohol intake (%)** |  |  |
| Excessive intake | 110270 (26.1) | 1748 (30.3) |
| No / moderate intake | 311292 (73.6) | 4000 (69.4) |
| Missing | 1303 (0.3) | 19 (0.3) |
| PM_2.5_ **(mean (SD)), µg/m3** | 10.00 (1.06) | 9.98 (1.07) |
| PM_10_ **(mean (SD)), µg/m3** | 19.31 (1.95) | 19.22 (1.90) |
| NO_2_ **(mean (SD)), µg/m3** | 29.32 (9.25) | 28.98 (9.09) |
| NO_X_ **(mean (SD)), µg/m3** | 44.09 (15.68) | 43.62 (15.73) |

^※^ TDI was dichotomized as low (≤-2.14) and high according to the median of TDI in non-cases.

* Sufficient physical activity was defined by at least ≥150 minutes moderate activity per week or ≥75 minutes vigorous activity per week (or an equivalent combination) according to the 2018 Physical Activity Guidelines for Americans.

**Supplementary Table 4. Baseline characteristics of study participants in CRC survival analysis.**

| **Characteristics** | **Non-death** | **All-cause death** |
| --- | --- | --- |
|  | (n=1868) | (n=533) |
| **Recruitment age (mean (SD))** | 61.51 (6.02) | 63.03 (5.43) |
| **Gender (%)** |  |  |
| Female | 870 (46.6) | 172 (32.3) |
| Male | 998 (53.4) | 361 (67.7) |
| **Education (%)** |  |  |
| College | 531 (28.4) | 114 (21.4) |
| Miss | 40 (2.1) | 15 (2.8) |
| Non-College | 1297 (69.4) | 404 (75.8) |
| **Townsend deprivation index (%)** | | |
| Low | 957 (51.2) | 244 (45.8) |
| High | 911 (48.8) | 289 (54.2) |
| **Family history of bowel cancer (%)** | | |
| No | 1162 (62.2) | 322 (60.4) |
| Yes | 276 (14.8) | 79 (14.8) |
| Missing | 430 (23.0) | 132 (24.8) |
| **Ethnicity (%)** |  |  |
| Non-White | 71 (3.8) | 15 (2.8) |
| White | 1791 (96.2) | 516 (97.2) |
| **BMI (%)** |  |  |
| Abnormal | 1322 (70.8) | 404 (75.8) |
| Normal | 534 (28.6) | 126 (23.6) |
| Missing | 12 (0.6) | 3 (0.6) |
| **Smoking status (%)** |  |  |
| Current / ever smoking | 976 (52.2) | 329 (61.7) |
| Never smoking | 875 (46.8) | 201 (37.7) |
| Missing | 17 (0.9) | 3 (0.6) |
| **Physical activity (%)** |  |  |
| Insufficient | 1333 (71.4) | 425 (79.7) |
| Sufficient | 397 (21.3) | 72 (13.5) |
| Missing | 138 (7.4) | 36 (6.8) |
| **Alcohol intake (%)** |  |  |
| Excessive intake | 451 (24.1) | 127 (23.8) |
| No / moderate intake | 1414 (75.7) | 403 (75.6) |
| Missing | 3 (0.2) | 3 (0.6) |
| PM_2.5_ **(mean (SD)), µg/m3** | 9.92 (1.09) | 10.08 (1.00) |
| PM_10_ **(mean (SD)), µg/m3** | 19.10 (1.94) | 19.31 (1.92) |
| NO_2_ **(mean (SD)), µg/m3** | 6.41 (0.92) | 6.41 (0.87) |
| NO_X_ **(mean (SD)), µg/m3** | 28.43 (9.13) | 29.58 (8.49) |

^※^ TDI was dichotomized as low (≤-1.92) and high according to the median of TDI in non-cases.

* Sufficient physical activity was defined by at least ≥150 minutes moderate activity per week or ≥75 minutes vigorous activity per week (or an equivalent combination) according to the 2018 Physical Activity Guidelines for Americans.

**Supplementary Table 5. Associations between individual and combined exposure to air pollution and risk of CRC incidence in UK Biobank, excluding participants with a reported family history of CRC.**

| **Pollution** | Cases | **Model 1** | |  | **Model 2** | |
| --- | --- | --- | --- | --- | --- | --- |
|  |  | HR (95% CI) | *P* value |  | HR (95% CI) | *P* value |
| **PM_2.5_ (μg/m3)** |  |  |  |  |  |  |
| Q1 | 1360 | 1.00 (ref) |  |  | 1.00 (ref) |  |
| Q2 | 1283 | 0.95 (0.88, 1.03) | 0.218 |  | 0.96 (0.89, 1.04) | 0.322 |
| Q3 | 1326 | 1.03 (0.95, 1.11) | 0.446 |  | 1.04 (0.96, 1.12) | 0.332 |
| Q4 | 1215 | 0.98 (0.91, 1.06) | 0.625 |  | 0.99 (0.91, 1.07) | 0.805 |
| P for trend |  |  | 0.998 |  |  | 0.840 |
| Per 5-μg/m3*day |  | 1.10 (0.85, 1.42) | 0.481 |  | 1.13 (0.85, 1.49) | 0.394 |
| **PM_10_ (μg/m3)** |  |  |  |  |  |  |
| Q1 | 1366 | 1.00 (ref) |  |  | 1.00 (ref) |  |
| Q2 | 1323 | 0.99 (0.91, 1.06) | 0.700 |  | 0.99 (0.92, 1.07) | 0.743 |
| Q3 | 1313 | 1.01 (0.93, 1.08) | 0.897 |  | 1.01 (0.93, 1.09) | 0.833 |
| Q4 | 1182 | 0.95 (0.88, 1.03) | 0.213 |  | 0.97 (0.90, 1.06) | 0.513 |
| P for trend |  |  | 0.270 |  |  | 0.612 |
| Per 5-μg/m3*day |  | 0.92 (0.80, 1.06) | 0.240 |  | 0.95 (0.82, 1.11) | 0.544 |
| **NO_2_ (μg/m3)** |  |  |  |  |  |  |
| Q1 | 1337 | 1.00 (ref) |  |  | 1.00 (ref) |  |
| Q2 | 1353 | 1.02 (0.95, 1.10) | 0.610 |  | 1.02 (0.95, 1.11) | 0.532 |
| Q3 | 1287 | 1.00 (0.93, 1.08) | 0.929 |  | 1.01 (0.94, 1.09) | 0.778 |
| Q4 | 1207 | 0.99 (0.92, 1.07) | 0.868 |  | 1.02 (0.94, 1.11) | 0.631 |
| P for trend |  |  | 0.768 |  |  | 0.702 |
| Per 5 μg/m3*day |  | 1.00 (0.97, 1.03) | 0.861 |  | 1.01 (0.97, 1.04) | 0.674 |
| **NO_x_ (μg/m3)** |  |  |  |  |  |  |
| Q1 | 1386 | 1.00 (ref) |  |  | 1.00 (ref) |  |
| Q2 | 1304 | 0.96 (0.89, 1.03) | 0.268 |  | 0.96 (0.89, 1.04) | 0.325 |
| Q3 | 1248 | 0.95 (0.88, 1.02) | 0.177 |  | 0.96 (0.89, 1.04) | 0.306 |
| Q4 | 1246 | 0.99 (0.92, 1.07) | 0.830 |  | 1.01 (0.93, 1.09) | 0.897 |
| P for trend |  |  | 0.812 |  |  | 0.916 |
| Per 5-μg/m3*day |  | 1.00 (0.98, 1.02) | 0.770 |  | 1.00 (0.98, 1.02) | 0.951 |
| **Combined air pollution exposure score** (per SD) | 5184 | 1.04 (1.01, 1.07) | 0.005 |  | 1.04 (1.01, 1.07) | 0.011 |

Model 1 adjusted for age at recruitment and sex.

Model 2 adjusted for age at recruitment, sex, education, Townsend deprivation index (TDI), ethnicity, BMI, alcohol intake, physical activity and smoking.

**Supplementary Table 6.** **Associations between individual and combined exposure to air pollution and risk of CRC incidence stratified by gender in UK Biobank.**

| Air pollutant | **Female** | | | |  | **Male** | | | | P interaction |
| --- | --- | --- | --- | --- | --- | --- | --- | --- | --- | --- |
|  | Model 1 | | Model 2 | |  | Model 1 | | Model 2 | |  |
|  | HR (95%CI) | P value | HR (95%CI) | P value |  | HR (95%CI) | P value | HR (95%CI) | P value |  |
| **PM_2.5_ (μg/m3)** |  |  |  |  |  |  |  |  |  | 0.279 |
| Q1 | 1.00 (Ref) |  | 1.00 (Ref) |  |  | 1.00 (Ref) |  | 1.00 (Ref) |  |  |
| Q2 | 1.01 (0.90, 1.13) | 0.874 | 1.01 (0.91, 1.13) | 0.794 |  | 0.94 (0.85, 1.03) | 0.199 | 0.95 (0.86, 1.04) | 0.276 |  |
| Q3 | 1.03 (0.92, 1.15) | 0.609 | 1.03 (0.92, 1.16) | 0.564 |  | 1.05 (0.95, 1.15) | 0.340 | 1.06 (0.96, 1.16) | 0.268 |  |
| Q4 | 1.05 (0.94, 1.17) | 0.385 | 1.04 (0.93, 1.17) | 0.482 |  | 0.97 (0.88, 1.07) | 0.527 | 0.98 (0.88, 1.09) | 0.686 |  |
| P for trend |  | 0.353 |  | 0.451 |  |  | 0.940 |  | 0.917 |  |
| Per 5-μg/m3*day | 1.37 (0.95, 1.98) | 0.096 | 1.35 (0.91, 2.01) | 0.138 |  | 1.02 (0.73, 1.41) | 0.917 | 1.04 (0.73, 1.47) | 0.845 |  |
| **PM_10_ (μg/m3)** |  |  |  |  |  |  |  |  |  | 0.636 |
| Q1 | 1.00 (Ref) |  | 1.00 (Ref) |  |  | 1.00 (Ref) |  | 1.00 (Ref) |  |  |
| Q2 | 0.99 (0.88, 1.10) | 0.817 | 0.99 (0.88, 1.10) | 0.828 |  | 1.00 (0.91, 1.10) | 0.974 | 1.01 (0.92, 1.11) | 0.888 |  |
| Q3 | 1.13 (1.02, 1.26) | 0.023 | 1.12 (1.01, 1.25) | 0.036 |  | 0.95 (0.86, 1.05) | 0.292 | 0.96 (0.87, 1.06) | 0.429 |  |
| Q4 | 1.00 (0.89, 1.12) | 0.995 | 1.00 (0.89, 1.13) | 0.993 |  | 0.96 (0.87, 1.05) | 0.368 | 0.99 (0.89, 1.10) | 0.838 |  |
| P for trend |  | 0.588 |  | 0.609 |  |  | 0.258 |  | 0.675 |  |
| Per 5-μg/m3*day | 1.04 (0.85, 1.27) | 0.701 | 1.03 (0.83, 1.28) | 0.771 |  | 0.90 (0.75, 1.07) | 0.232 | 0.95 (0.79, 1.15) | 0.620 |  |
| **NO_2_ (μg/m3)** |  |  |  |  |  |  |  |  |  | 0.495 |
| Q1 | 1.00 (Ref) |  | 1.00 (Ref) |  |  | 1.00 (Ref) |  | 1.00 (Ref) |  |  |
| Q2 | 1.01 (0.90, 1.13) | 0.875 | 1.02 (0.91, 1.14) | 0.748 |  | 1.00 (0.91, 1.10) | 0.954 | 1.00 (0.91, 1.10) | 0.968 |  |
| Q3 | 1.04 (0.93, 1.16) | 0.472 | 1.04 (0.93, 1.16) | 0.492 |  | 0.98 (0.89, 1.08) | 0.676 | 0.99 (0.90, 1.09) | 0.843 |  |
| Q4 | 1.02 (0.91, 1.14) | 0.744 | 1.01 (0.89, 1.14) | 0.862 |  | 1.00 (0.90, 1.10) | 0.926 | 1.04 (0.93, 1.16) | 0.483 |  |
| P for trend |  | 0.663 |  | 0.801 |  |  | 0.867 |  | 0.524 |  |
| Per 5-μg/m3*day | 1.01 (0.97, 1.06) | 0.564 | 1.01 (0.96, 1.06) | 0.767 |  | 1.00 (0.96, 1.04) | 0.917 | 1.01 (0.97, 1.06) | 0.512 |  |
| **NO_X_ (μg/m3)** |  |  |  |  |  |  |  |  |  | 0.221 |
| Q1 | 1.00 (Ref) |  | 1.00 (Ref) |  |  | 1.00 (Ref) |  | 1.00 (Ref) |  |  |
| Q2 | 0.94 (0.84, 1.04) | 0.240 | 0.94 (0.84, 1.05) | 0.269 |  | 0.99 (0.90, 1.09) | 0.845 | 0.99 (0.90, 1.09) | 0.899 |  |
| Q3 | 0.96 (0.86, 1.07) | 0.469 | 0.96 (0.86, 1.08) | 0.493 |  | 0.95 (0.86, 1.05) | 0.289 | 0.96 (0.87, 1.06) | 0.424 |  |
| Q4 | 1.04 (0.93, 1.16) | 0.491 | 1.03 (0.92, 1.16) | 0.601 |  | 0.99 (0.90, 1.09) | 0.825 | 1.01 (0.91, 1.12) | 0.898 |  |
| P for trend |  | 0.412 |  | 0.547 |  |  | 0.681 |  | 0.975 |  |
| Per 5-μg/m3*day | 1.01 (0.99, 1.04) | 0.375 | 1.01 (0.98, 1.04) | 0.469 |  | 1.00 (0.97, 1.02) | 0.714 | 1.00 (0.97, 1.02) | 0.878 |  |
| **Combined air pollution exposure score (per SD)** | 1.00 (1.00, 1.01) | 0.401 | 1.00 (0.99, 1.01) | 0.519 |  | 1.01 (1.00, 1.01) | 0.008 | 1.01 (1.00, 1.01) | 0.011 | 0.387 |

Model 1 adjusted for age at recruitment.

Model 2 adjusted for age at recruitment, education, Townsend deprivation index (TDI), ethnicity, family history of CRC, BMI, alcohol intake, physical activity, and smoking.

**Supplementary Table 7.** **Associations between individual and combined exposure to air pollution and risk of CRC incidence stratified by smoking status in UK Biobank.**

| Air pollutant | **Currently or ever smoking** | | | |  | **Never smoking** | | | | P interaction |
| --- | --- | --- | --- | --- | --- | --- | --- | --- | --- | --- |
|  | Model 1 | | Model 2 | |  | Model 1 | | Model 2 | |  |
|  | HR (95%CI) | P value | HR (95%CI) | P value |  | HR (95%CI) | P value | HR (95%CI) | P value |  |
| **PM_2.5_ (μg/m3)** |  |  |  |  |  |  |  |  |  | 0.427 |
| Q1 | 1.00 (Ref) |  | 1.00 (Ref) |  |  | 1.00 (Ref) |  | 1.00 (Ref) |  |  |
| Q2 | 0.94 (0.85, 1.03) | 0.199 | 0.94 (0.85, 1.04) | 0.266 |  | 1.01 (0.91, 1.12) | 0.916 | 1.02 (0.91, 1.13) | 0.773 |  |
| Q3 | 1.05 (0.95, 1.15) | 0.340 | 1.00 (0.91, 1.11) | 0.959 |  | 1.09 (0.98, 1.21) | 0.116 | 1.10 (0.99, 1.23) | 0.072 |  |
| Q4 | 0.97 (0.88, 1.07) | 0.527 | 0.97 (0.87, 1.08) | 0.564 |  | 1.04 (0.93, 1.16) | 0.523 | 1.06 (0.94, 1.19) | 0.329 |  |
| P for trend |  | 0.940 |  | 0.765 |  |  | 0.314 |  | 0.182 |  |
| Per 5-μg/m3*day | 1.02 (0.73, 1.41) | 0.917 | 0.98 (0.69, 1.40) | 0.919 |  | 1.35 (0.94, 1.95) | 0.108 | 1.49 (1.00, 2.20) | 0.047 |  |
| **PM_10_ (μg/m3)** |  |  |  |  |  |  |  |  |  | 0.169 |
| Q1 | 1.00 (Ref) |  | 1.00 (Ref) |  |  | 1.00 (Ref) |  | 1.00 (Ref) |  |  |
| Q2 | 1.00 (0.91, 1.10) | 0.974 | 1.00 (0.91, 1.11) | 0.965 |  | 0.98 (0.89, 1.09) | 0.760 | 0.99 (0.89, 1.10) | 0.844 |  |
| Q3 | 0.95 (0.86, 1.05) | 0.292 | 0.99 (0.90, 1.10) | 0.863 |  | 1.07 (0.96, 1.19) | 0.202 | 1.08 (0.97, 1.20) | 0.151 |  |
| Q4 | 0.96 (0.87, 1.05) | 0.368 | 0.99 (0.89, 1.11) | 0.924 |  | 0.96 (0.86, 1.07) | 0.471 | 0.99 (0.88, 1.11) | 0.869 |  |
| P for trend |  | 0.258 |  | 0.887 |  |  | 0.749 |  | 0.801 |  |
| Per 5-μg/m3*day | 0.90 (0.75, 1.07) | 0.232 | 0.96 (0.79, 1.17) | 0.672 |  | 0.96 (0.79, 1.18) | 0.723 | 1.03 (0.83, 1.27) | 0.817 |  |
| **NO_2_ (μg/m3)** |  |  |  |  |  |  |  |  |  | 0.396 |
| Q1 | 1.00 (Ref) |  | 1.00 (Ref) |  |  | 1.00 (Ref) |  | 1.00 (Ref) |  |  |
| Q2 | 1.00 (0.91, 1.10) | 0.954 | 0.93 (0.84, 1.02) | 0.138 |  | 1.10 (0.99, 1.22) | 0.066 | 1.11 (1.00, 1.23) | 0.052 |  |
| Q3 | 0.98 (0.89, 1.08) | 0.676 | 0.95 (0.86, 1.05) | 0.304 |  | 1.08 (0.97, 1.20) | 0.176 | 1.09 (0.98, 1.21) | 0.124 |  |
| Q4 | 1.00 (0.90, 1.10) | 0.926 | 0.97 (0.87, 1.08) | 0.607 |  | 1.05 (0.94, 1.17) | 0.378 | 1.10 (0.97, 1.24) | 0.136 |  |
| P for trend |  | 0.867 |  | 0.730 |  |  | 0.492 |  | 0.165 |  |
| Per 5-μg/m3*day | 1.00 (0.96, 1.04) | 0.917 | 1.00 (0.96, 1.04) | 0.916 |  | 1.01 (0.97, 1.06) | 0.513 | 1.03 (0.98, 1.08) | 0.191 |  |
| **NO_X_ (μg/m3)** |  |  |  |  |  |  |  |  |  | 0.853 |
| Q1 | 1.00 (Ref) |  | 1.00 (Ref) |  |  | 1.00 (Ref) |  | 1.00 (Ref) |  |  |
| Q2 | 0.99 (0.90, 1.09) | 0.845 | 0.90 (0.82, 1.00) | 0.046 |  | 1.04 (0.94, 1.16) | 0.417 | 1.05 (0.95, 1.17) | 0.349 |  |
| Q3 | 0.95 (0.86, 1.05) | 0.289 | 0.91 (0.82, 1.01) | 0.066 |  | 1.01 (0.91, 1.12) | 0.857 | 1.03 (0.92, 1.15) | 0.626 |  |
| Q4 | 0.99 (0.90, 1.09) | 0.825 | 0.95 (0.86, 1.06) | 0.386 |  | 1.06 (0.95, 1.19) | 0.264 | 1.10 (0.98, 1.23) | 0.118 |  |
| P for trend |  | 0.681 |  | 0.449 |  |  | 0.351 |  | 0.157 |  |
| Per 5-μg/m3*day | 1.00 (0.97, 1.02) | 0.714 | 1.00 (0.97, 1.02) | 0.688 |  | 1.01 (0.98, 1.03) | 0.475 | 1.02 (0.99, 1.04) | 0.261 |  |
| **Combined air pollution exposure score (per SD)** | 1.05 (1.01, 1.09) | 0.006 | 1.05 (1.01, 1.08) | 0.018 |  | 1.03 (0.99, 1.07) | 0.188 | 1.02 (0.98, 1.07) | 0.308 | 0.514 |

Model 1 adjusted for age at recruitment and sex.

Model 2 adjusted for age at recruitment, sex, education, Townsend deprivation index (TDI), ethnicity, family history of CRC, BMI, alcohol intake and physical activity.

**Supplementary Table 8.** **Associations between individual and combined exposure to air pollution and risk of CRC incidence stratified by physical activity in UK Biobank.**

| Air pollutant | **Insufficient physical activity** | | | |  | **Sufficient physical activity*** | | | | P interaction |
| --- | --- | --- | --- | --- | --- | --- | --- | --- | --- | --- |
|  | Model 1 | | Model 2 | |  | Model 1 | | Model 2 | |  |
|  | HR (95%CI) | P value | HR (95%CI) | P value |  | HR (95%CI) | P value | HR (95%CI) | P value |  |
| **PM_2.5_ (μg/m3)** |  |  |  |  |  |  |  |  |  | 0.152 |
| Q1 | 1.00 (Ref) |  | 1.00 (Ref) |  |  | 1.00 (Ref) |  | 1.00 (Ref) |  |  |
| Q2 | 0.98 (0.90, 1.07) | 0.692 | 1.00 (0.91, 1.09) | 0.923 |  | 0.93 (0.80, 1.09) | 0.383 | 0.94 (0.81, 1.10) | 0.431 |  |
| Q3 | 1.07 (0.98, 1.16) | 0.132 | 1.08 (0.99, 1.18) | 0.069 |  | 0.98 (0.84, 1.14) | 0.770 | 0.98 (0.84, 1.15) | 0.791 |  |
| Q4 | 0.99 (0.91, 1.08) | 0.886 | 1.01 (0.92, 1.11) | 0.785 |  | 1.06 (0.91, 1.24) | 0.470 | 1.05 (0.89, 1.24) | 0.543 |  |
| P for trend |  | 0.745 |  | 0.472 |  |  | 0.451 |  | 0.537 |  |
| Per 5-μg/m3*day | 1.12 (0.84, 1.50) | 0.443 | 1.20 (0.88, 1.64) | 0.245 |  | 1.23 (0.72, 2.11) | 0.441 | 1.20 (0.68, 2.14) | 0.525 |  |
| **PM_10_ (μg/m3)** |  |  |  |  |  |  |  |  |  | 0.969 |
| Q1 | 1.00 (Ref) |  | 1.00 (Ref) |  |  | 1.00 (Ref) |  | 1.00 (Ref) |  |  |
| Q2 | 0.97 (0.89, 1.06) | 0.535 | 0.98 (0.90, 1.07) | 0.660 |  | 1.06 (0.91, 1.23) | 0.470 | 1.06 (0.91, 1.24) | 0.432 |  |
| Q3 | 1.01 (0.92, 1.09) | 0.906 | 1.02 (0.93, 1.11) | 0.713 |  | 1.07 (0.92, 1.25) | 0.369 | 1.07 (0.92, 1.26) | 0.372 |  |
| Q4 | 0.96 (0.88, 1.05) | 0.334 | 0.99 (0.90, 1.08) | 0.802 |  | 1.01 (0.86, 1.18) | 0.939 | 1.01 (0.86, 1.20) | 0.867 |  |
| P for trend |  | 0.441 |  | 0.957 |  |  | 0.884 |  | 0.816 |  |
| Per 5-μg/m3*day | 0.92 (0.79, 1.08) | 0.309 | 0.98 (0.83, 1.16) | 0.805 |  | 1.01 (0.75, 1.35) | 0.942 | 1.02 (0.75, 1.40) | 0.880 |  |
| **NO_2_ (μg/m3)** |  |  |  |  |  |  |  |  |  | 0.547 |
| Q1 | 1.00 (Ref) |  | 1.00 (Ref) |  |  | 1.00 (Ref) |  | 1.00 (Ref) |  |  |
| Q2 | 0.98 (0.90, 1.06) | 0.597 | 0.98 (0.90, 1.07) | 0.719 |  | 1.04 (0.90, 1.22) | 0.576 | 1.04 (0.90, 1.22) | 0.576 |  |
| Q3 | 0.99 (0.91, 1.08) | 0.819 | 1.00 (0.92, 1.09) | 0.981 |  | 1.06 (0.91, 1.24) | 0.461 | 1.06 (0.91, 1.24) | 0.465 |  |
| Q4 | 0.97 (0.89, 1.06) | 0.527 | 1.01 (0.92, 1.11) | 0.880 |  | 1.10 (0.93, 1.29) | 0.256 | 1.11 (0.93, 1.33) | 0.229 |  |
| P for trend |  | 0.596 |  | 0.812 |  |  | 0.251 |  | 0.228 |  |
| Per 5-μg/m3*day | 1.00 (0.97, 1.03) | 0.936 | 1.01 (0.98, 1.05) | 0.456 |  | 1.01 (0.95, 1.08) | 0.708 | 1.01 (0.95, 1.09) | 0.682 |  |
| **NO_X_ (μg/m3)** |  |  |  |  |  |  |  |  |  | 0.088 |
| Q1 | 1.00 (Ref) |  | 1.00 (Ref) |  |  | 1.00 (Ref) |  | 1.00 (Ref) |  |  |
| Q2 | 0.97 (0.89, 1.05) | 0.443 | 0.97 (0.89, 1.06) | 0.555 |  | 0.97 (0.83, 1.13) | 0.680 | 0.97 (0.83, 1.13) | 0.670 |  |
| Q3 | 0.98 (0.90, 1.06) | 0.569 | 0.99 (0.91, 1.08) | 0.856 |  | 0.92 (0.79, 1.08) | 0.318 | 0.92 (0.78, 1.08) | 0.315 |  |
| Q4 | 1.00 (0.92, 1.09) | 0.929 | 1.03 (0.94, 1.13) | 0.540 |  | 1.02 (0.87, 1.19) | 0.815 | 1.01 (0.85, 1.19) | 0.943 |  |
| P for trend |  | 0.875 |  | 0.497 |  |  | 0.965 |  | 0.908 |  |
| Per 5-μg/m3*day | 1.00 (0.98, 1.02) | 0.943 | 1.01 (0.99, 1.03) | 0.580 |  | 1.01 (0.97, 1.05) | 0.591 | 1.01 (0.97, 1.05) | 0.670 |  |
| **Combined air pollution exposure score (per SD)** | 1.05 (1.01, 1.09) | 0.009 | 1.05 (1.01, 1.08) | 0.014 |  | 1.01 (0.96, 1.06) | 0.838 | 1.01 (0.96, 1.06) | 0.830 | 0.197 |

Model 1 adjusted for age at recruitment and sex.

Model 2 adjusted for age at recruitment, sex, education, Townsend deprivation index (TDI), ethnicity, family history of CRC, BMI, alcohol intake, and smoking.

* Sufficient physical activity was defined by at least ≥150 minutes moderate activity per week or ≥75 minutes vigorous activity per week (or an equivalent combination) according to the 2018 Physical Activity Guidelines for Americans (DOI: 10.1001/jama.2018.14854).

**Supplementary Table 9.** **Associations between individual and combined exposure to air pollution and risk of CRC incidence stratified by anatomic sites in UK Biobank.**

| Air pollutant | **Colon** | | | |  | **Rectum** | | | |
| --- | --- | --- | --- | --- | --- | --- | --- | --- | --- |
|  | Model 1 | | Model 2 | |  | Model 1 | | Model 2 | |
|  | HR (95%CI) | P value | HR (95%CI) | P value |  | HR (95%CI) | P value | HR (95%CI) | P value |
| **PM_2.5_ (μg/m3)** |  |  |  |  |  |  |  |  |  |
| Q1 | 1.00 (Ref) |  | 1.00 (Ref) |  |  | 1.00 (Ref) |  | 1.00 (Ref) |  |
| Q2 | 0.96 (0.88, 1.06) | 0.445 | 0.97 (0.88, 1.07) | 0.541 |  | 0.94 (0.81, 1.09) | 0.402 | 0.95 (0.82, 1.10) | 0.482 |
| Q3 | 1.03 (0.94, 1.13) | 0.492 | 1.04 (0.95, 1.15) | 0.394 |  | 0.95 (0.82, 1.10) | 0.484 | 0.95 (0.82, 1.10) | 0.486 |
| Q4 | 0.98 (0.89, 1.08) | 0.710 | 0.98 (0.89, 1.09) | 0.762 |  | 0.94 (0.81, 1.09) | 0.382 | 0.93 (0.79, 1.09) | 0.369 |
| P for trend |  | 0.992 |  | 0.964 |  |  | 0.409 |  | 0.378 |
| Per 5-μg/m3*day | 1.15 (0.84, 1.58) | 0.393 | 1.17 (0.83, 1.65) | 0.366 |  | 0.87 (0.53, 1.44) | 0.590 | 0.83 (0.48, 1.42) | 0.489 |
| **PM_10_ (μg/m3)** |  |  |  |  |  |  |  |  |  |
| Q1 | 1.00 (Ref) |  | 1.00 (Ref) |  |  | 1.00 (Ref) |  | 1.00 (Ref) |  |
| Q2 | 1.00 (0.91, 1.10) | 0.983 | 1.00 (0.91, 1.10) | 0.961 |  | 0.96 (0.84, 1.11) | 0.622 | 0.97 (0.84, 1.12) | 0.671 |
| Q3 | 1.03 (0.94, 1.13) | 0.496 | 1.03 (0.94, 1.13) | 0.532 |  | 0.93 (0.81, 1.08) | 0.340 | 0.94 (0.81, 1.09) | 0.423 |
| Q4 | 0.95 (0.86, 1.04) | 0.278 | 0.96 (0.87, 1.06) | 0.417 |  | 0.90 (0.77, 1.04) | 0.157 | 0.93 (0.79, 1.08) | 0.334 |
| P for trend |  | 0.361 |  | 0.516 |  |  | 0.138 |  | 0.306 |
| Per 5-μg/m3*day | 0.92 (0.77, 1.09) | 0.340 | 0.93 (0.77, 1.12) | 0.461 |  | 0.80 (0.61, 1.05) | 0.115 | 0.84 (0.63, 1.13) | 0.249 |
| **NO_2_ (μg/m3)** |  |  |  |  |  |  |  |  |  |
| Q1 | 1.00 (Ref) |  | 1.00 (Ref) |  |  | 1.00 (Ref) |  | 1.00 (Ref) |  |
| Q2 | 1.02 (0.93, 1.11) | 0.718 | 1.02 (0.93, 1.12) | 0.664 |  | 0.94 (0.81, 1.09) | 0.392 | 0.94 (0.81, 1.09) | 0.427 |
| Q3 | 1.00 (0.91, 1.09) | 0.942 | 1.00 (0.91, 1.10) | 0.931 |  | 0.95 (0.82, 1.10) | 0.460 | 0.96 (0.83, 1.11) | 0.569 |
| Q4 | 0.94 (0.85, 1.03) | 0.174 | 0.93 (0.84, 1.04) | 0.192 |  | 0.98 (0.84, 1.13) | 0.748 | 1.02 (0.87, 1.20) | 0.810 |
| P for trend |  | 0.142 |  | 0.171 |  |  | 0.815 |  | 0.773 |
| Per 5-μg/m3*day | 0.97 (0.94, 1.01) | 0.130 | 0.97 (0.93, 1.01) | 0.117 |  | 1.01 (0.95, 1.07) | 0.758 | 1.03 (0.96, 1.10) | 0.395 |
| **NO_X_ (μg/m3)** |  |  |  |  |  |  |  |  |  |
| Q1 | 1.00 (Ref) |  | 1.00 (Ref) |  |  | 1.00 (Ref) |  | 1.00 (Ref) |  |
| Q2 | 0.95 (0.87, 1.05) | 0.317 | 0.96 (0.87, 1.05) | 0.363 |  | 0.97 (0.84, 1.12) | 0.657 | 0.97 (0.84, 1.12) | 0.668 |
| Q3 | 0.94 (0.85, 1.03) | 0.184 | 0.94 (0.86, 1.04) | 0.241 |  | 0.92 (0.79, 1.06) | 0.236 | 0.92 (0.79, 1.07) | 0.272 |
| Q4 | 1.00 (0.91, 1.10) | 0.950 | 1.00 (0.90, 1.11) | 0.977 |  | 0.92 (0.79, 1.06) | 0.243 | 0.91 (0.78, 1.07) | 0.265 |
| P for trend | 1.00 (1.00, 1.00) | 0.906 | 1.00 (1.00, 1.00) | 0.963 |  | 1.00 (0.99, 1.00) | 0.190 | 1.00 (0.99, 1.00) | 0.218 |
| Per 5-μg/m3*day |  | 0.875 |  | 0.871 |  |  | 0.111 |  | 0.114 |
| **Combined air pollution exposure score (per SD)** | 1.04 (1.00, 1.08) | 0.027 | 1.04 (1.00, 1.08) | 0.044 |  | 1.05 (1.00, 1.11) | 0.072 | 1.05 (0.99, 1.11) | 0.094 |

Model 1 adjusted for age at recruitment and sex.

Model 2 adjusted for age at recruitment, sex, education, Townsend deprivation index (TDI), ethnicity, family history of CRC, BMI, alcohol intake, and smoking.

**Supplementary Table 10. Associations between air pollution and risk of all-cause mortality from CRC diagnosed within 5 years.**

| **Pollution** | Cases | **Model 1** | |  | **Model 2** | |
| --- | --- | --- | --- | --- | --- | --- |
|  |  | HR (95% CI) | *P* value |  | HR (95% CI) | *P* value |
| **PM_2.5_ (μg/m3)** |  |  |  |  |  |  |
| Q1 | 59 | 1.00 (ref) |  |  | 1.00 (ref) |  |
| Q2 | 78 | 1.40 (1.08, 1.80) | 0.010 |  | 1.34 (1.04, 1.73) | 0.024 |
| Q3 | 86 | 1.42 (1.10, 1.84) | 0.007 |  | 1.35 (1.04, 1.76) | 0.023 |
| Q4 | 105 | 1.56 (1.21, 2.00) | 0.001 |  | 1.43 (1.10, 1.87) | 0.008 |
| P for trend |  |  | 0.001 |  |  | 0.011 |
| Per 5-μg/m3*day |  | 1.77 (1.23, 2.55) | 0.002 |  | 1.54 (1.04, 2.29) | 0.031 |
| **PM_10_ (μg/m3)** |  |  |  |  |  |  |
| Q1 | 69 | 1.00 (ref) |  |  | 1.00 (ref) |  |
| Q2 | 82 | 1.05 (0.82, 1.34) | 0.712 |  | 1.02 (0.79, 1.3) | 0.903 |
| Q3 | 82 | 1.10 (0.86, 1.41) | 0.427 |  | 1.04 (0.81, 1.33) | 0.758 |
| Q4 | 95 | 1.27 (1.00, 1.61) | 0.051 |  | 1.19 (0.93, 1.53) | 0.172 |
| P for trend |  |  | 0.042 |  |  | 0.156 |
| Per 5-μg/m3*day |  | 1.34 (1.08, 1.67) | 0.008 |  | 1.28 (1.02, 1.62) | 0.037 |
| **NO_2_ (μg/m3)** |  |  |  |  |  |  |
| Q1 | 58 | 1.00 (ref) |  |  | 1.00 (ref) |  |
| Q2 | 82 | 1.50 (1.16, 1.94) | 0.002 |  | 1.45 (1.12, 1.88) | 0.005 |
| Q3 | 90 | 1.61 (1.25, 2.08) | 0.000 |  | 1.54 (1.19, 2.00) | 0.001 |
| Q4 | 98 | 1.62 (1.25, 2.09) | 0.000 |  | 1.52 (1.16, 2.00) | 0.003 |
| P for trend |  |  | 0.000 |  |  | 0.005 |
| Per 5 μg/m3*day |  | 1.07 (1.03, 1.12) | 0.002 |  | 1.06 (1.01, 1.12) | 0.016 |
| **NO_X_ (μg/m3)** |  |  |  |  |  |  |
| Q1 | 60 | 1.00 (ref) |  |  | 1.00 (ref) |  |
| Q2 | 72 | 1.28 (0.99, 1.66) | 0.056 |  | 1.25 (0.97, 1.62) | 0.089 |
| Q3 | 92 | 1.42 (1.11, 1.83) | 0.006 |  | 1.35 (1.04, 1.75) | 0.023 |
| Q4 | 104 | 1.53 (1.19, 1.96) | 0.001 |  | 1.43 (1.10, 1.86) | 0.008 |
| P for trend |  |  | 0.001 |  |  | 0.008 |
| Per 5-μg/m3*day |  | 1.04 (1.01, 1.07) | 0.003 |  | 1.03 (1.00, 1.06) | 0.027 |
| **Combined air pollution exposure score** (per SD increment) | 328 | 1.17 (1.06, 1.29) | 0.003 |  | 1.20 (1.06, 1.34) | 0.003 |

Model 1 adjusted for age at recruitment and sex.

Model 2 adjusted for age at recruitment, sex, education, TDI, ethnicity, family history of CRC, BMI, alcohol intake, physical activity and smoking.

**Supplementary Table 11. Associations between individual and combined exposure to air pollution and risk of all-cause mortality among CRC survivors in UK Biobank , excluding participants with a reported family history of CRC.**

| **Pollution** | Cases | **Model 1** | |  | **Model 2** | |
| --- | --- | --- | --- | --- | --- | --- |
|  |  | HR (95% CI) | *P* value |  | HR (95% CI) | *P* value |
| **PM_2.5_ (μg/m3)** |  |  |  |  |  |  |
| Q1 | 87 | 1.00 (ref) |  |  | 1.00 (ref) |  |
| Q2 | 122 | 1.42 (1.08, 1.87) | 0.013 |  | 1.39 (1.05, 1.83) | 0.021 |
| Q3 | 113 | 1.47 (1.11, 1.95) | 0.007 |  | 1.42 (1.07, 1.89) | 0.017 |
| Q4 | 132 | 1.61 (1.23, 2.11) | 0.001 |  | 1.51 (1.14, 2.02) | 0.004 |
| P for trend |  |  | 0.001 |  |  | 0.006 |
| Per 5-μg/m3*day |  | 1.85 (1.25, 2.74) | 0.002 |  | 1.66 (1.09, 2.54) | 0.019 |
| **PM_10_ (μg/m3)** |  |  |  |  |  |  |
| Q1 | 107 | 1.00 (ref) |  |  | 1.00 (ref) |  |
| Q2 | 112 | 1.08 (0.83, 1.40) | 0.580 |  | 1.02 (0.78, 1.34) | 0.858 |
| Q3 | 107 | 1.09 (0.84, 1.43) | 0.508 |  | 1.03 (0.79, 1.36) | 0.805 |
| Q4 | 128 | 1.31 (1.02, 1.70) | 0.038 |  | 1.24 (0.95, 1.62) | 0.118 |
| P for trend |  |  | 0.036 |  |  | 0.110 |
| Per 5-μg/m3*day |  | 1.34 (1.06, 1.69) | 0.013 |  | 1.30 (1.01, 1.67) | 0.041 |
| **NO_2_ (μg/m3)** |  |  |  |  |  |  |
| Q1 | 86 | 1.00 (ref) |  |  | 1.00 (ref) |  |
| Q2 | 121 | 1.51 (1.15, 2.00) | 0.003 |  | 1.45 (1.10, 1.92) | 0.009 |
| Q3 | 120 | 1.51 (1.15, 1.99) | 0.003 |  | 1.45 (1.09, 1.92) | 0.010 |
| Q4 | 127 | 1.65 (1.25, 2.16) | 0.000 |  | 1.58 (1.18, 2.12) | 0.002 |
| P for trend |  |  | 0.001 |  |  | 0.004 |
| Per 5 μg/m3*day |  | 1.07 (1.02, 1.12) | 0.004 |  | 1.07 (1.01, 1.13) | 0.014 |
| **NO_x_ (μg/m3)** |  |  |  |  |  |  |
| Q1 | 93 | 1.00 (ref) |  |  | 1.00 (ref) |  |
| Q2 | 105 | 1.23 (0.93, 1.63) | 0.141 |  | 1.19 (0.90, 1.58) | 0.215 |
| Q3 | 122 | 1.36 (1.04, 1.78) | 0.026 |  | 1.31 (0.99, 1.72) | 0.057 |
| Q4 | 134 | 1.57 (1.21, 2.05) | 0.001 |  | 1.49 (1.13, 1.97) | 0.005 |
| P for trend |  |  | 0.001 |  |  | 0.004 |
| Per 5-μg/m3*day |  | 1.04 (1.01, 1.07) | 0.005 |  | 1.03 (1.00, 1.06) | 0.024 |
| **Combined air pollution exposure score** (per SD) | 454 | 1.15 (1.05, 1.25) | 0.002 |  | 1.14 (1.03, 1.25) | 0.009 |

Model 1 adjusted for age at recruitment and sex.

Model 2 adjusted for age at recruitment, sex, education, TDI, ethnicity, BMI, alcohol intake, physical activity and smoking.

**Supplementary Table 12.** **Associations between individual and combined exposure to air pollution and risk of all-cause mortality in CRC survivors stratified by gender in UK Biobank.**

| Air pollutant | **Female** | | | |  | **Male** | | | | P interaction |
| --- | --- | --- | --- | --- | --- | --- | --- | --- | --- | --- |
|  | Model 1 | | Model 2 | |  | Model 1 | | Model 2 | |  |
|  | HR (95%CI) | P value | HR (95%CI) | P value |  | HR (95%CI) | P value | HR (95%CI) | P value |  |
| **PM_2.5_ (μg/m3)** |  |  |  |  |  |  |  |  |  | 0.279 |
| Q1 | 1.00 (Ref) |  | 1.00 (Ref) |  |  | 1.00 (Ref) |  | 1.00 (Ref) |  |  |
| Q2 | 1.42 (0.92, 2.19) | 0.109 | 1.41 (0.91, 2.17) | 0.124 |  | 1.38 (1.01, 1.89) | 0.043 | 1.28 (0.93, 1.76) | 0.126 |  |
| Q3 | 1.29 (0.84, 1.99) | 0.250 | 1.26 (0.81, 1.96) | 0.307 |  | 1.50 (1.09, 2.05) | 0.012 | 1.38 (1.00, 1.91) | 0.052 |  |
| Q4 | 1.32 (0.84, 2.07) | 0.224 | 1.39 (0.87, 2.23) | 0.172 |  | 1.66 (1.23, 2.25) | 0.001 | 1.43 (1.03, 1.97) | 0.031 |  |
| P for trend |  | 0.277 |  | 0.217 |  |  | 0.001 |  | 0.034 |  |
| Per 5-μg/m3*day | 1.12 (0.55, 2.30) | 0.753 | 1.22 (0.56, 2.64) | 0.612 |  | 2.08 (1.37, 3.15) | 0.001 | 1.64 (1.04, 2.59) | 0.033 |  |
| **PM_10_ (μg/m3)** |  |  |  |  |  |  |  |  |  | 0.636 |
| Q1 | 1.00 (Ref) |  | 1.00 (Ref) |  |  | 1.00 (Ref) |  | 1.00 (Ref) |  |  |
| Q2 | 0.90 (0.58, 1.39) | 0.637 | 0.90 (0.58, 1.39) | 0.629 |  | 1.12 (0.83, 1.51) | 0.450 | 1.06 (0.79, 1.44) | 0.691 |  |
| Q3 | 1.01 (0.66, 1.54) | 0.980 | 0.98 (0.63, 1.51) | 0.920 |  | 1.15 (0.86, 1.56) | 0.347 | 1.03 (0.76, 1.40) | 0.847 |  |
| Q4 | 1.13 (0.74, 1.71) | 0.568 | 1.22 (0.79, 1.88) | 0.377 |  | 1.34 (1.00, 1.80) | 0.049 | 1.18 (0.87, 1.60) | 0.297 |  |
| P for trend |  | 0.461 |  | 0.316 |  |  | 0.049 |  | 0.321 |  |
| Per 5-μg/m3*day | 1.14 (0.78, 1.68) | 0.499 | 1.25 (0.82, 1.91) | 0.294 |  | 1.45 (1.11, 1.88) | 0.006 | 1.31 (0.98, 1.74) | 0.067 |  |
| **NO_2_ (μg/m3)** |  |  |  |  |  |  |  |  |  | 0.495 |
| Q1 | 1.00 (Ref) |  | 1.00 (Ref) |  |  | 1.00 (Ref) |  | 1.00 (Ref) |  |  |
| Q2 | 1.16 (0.74, 1.83) | 0.513 | 1.14 (0.72, 1.81) | 0.562 |  | 1.69 (1.24, 2.32) | 0.001 | 1.58 (1.15, 2.17) | 0.005 |  |
| Q3 | 1.52 (0.99, 2.33) | 0.055 | 1.51 (0.98, 2.33) | 0.063 |  | 1.66 (1.21, 2.27) | 0.002 | 1.53 (1.10, 2.11) | 0.011 |  |
| Q4 | 1.32 (0.85, 2.06) | 0.219 | 1.43 (0.89, 2.29) | 0.135 |  | 1.78 (1.30, 2.44) | 0.000 | 1.53 (1.09, 2.14) | 0.014 |  |
| P for trend |  | 0.157 |  | 0.086 |  |  | 0.001 |  | 0.040 |  |
| Per 5-μg/m3*day | 1.04 (0.96, 1.12) | 0.368 | 1.06 (0.97, 1.16) | 0.171 |  | 1.09 (1.03, 1.15) | 0.002 | 1.06 (1.00, 1.13) | 0.052 |  |
| **NO_X_ (μg/m3)** |  |  |  |  |  |  |  |  |  | 0.221 |
| Q1 | 1.00 (Ref) |  | 1.00 (Ref) |  |  | 1.00 (Ref) |  | 1.00 (Ref) |  |  |
| Q2 | 1.22 (0.79, 1.89) | 0.365 | 1.18 (0.76, 1.83) | 0.461 |  | 1.33 (0.97, 1.82) | 0.082 | 1.28 (0.93, 1.76) | 0.131 |  |
| Q3 | 1.44 (0.95, 2.17) | 0.088 | 1.41 (0.92, 2.15) | 0.117 |  | 1.42 (1.04, 1.95) | 0.029 | 1.30 (0.94, 1.79) | 0.118 |  |
| Q4 | 1.18 (0.75, 1.84) | 0.479 | 1.28 (0.80, 2.04) | 0.309 |  | 1.71 (1.27, 2.32) | 0.000 | 1.49 (1.08, 2.06) | 0.016 |  |
| P for trend |  | 0.352 |  | 0.216 |  |  | 0.000 |  | 0.020 |  |
| Per 5-μg/m3*day | 1.00 (0.96, 1.05) | 0.882 | 1.01 (0.96, 1.07) | 0.662 |  | 1.06 (1.03, 1.09) | 0.000 | 1.04 (1.01, 1.08) | 0.016 |  |
| **Combined air pollution exposure score (per SD)** | 1.03 (0.89, 1.20) | 0.698 | 1.07 (0.90, 1.26) | 0.451 |  | 1.19 (1.08, 1.32) | 0.001 | 1.15 (1.02, 1.28) | 0.018 | 0.109 |

Model 1 adjusted for age at recruitment.

Model 2 adjusted for age at recruitment, education, Townsend deprivation index (TDI), ethnicity, family history of CRC, BMI, alcohol intake, physical activity, and smoking.

**Supplementary Table 13.** **Associations between individual and combined exposure to air pollution and risk of all-cause mortality in CRC survivors stratified by smoking status in UK Biobank.**

| Air pollutant | **Currently or ever smoking** | | | |  | **Never smoking** | | | | P interaction |
| --- | --- | --- | --- | --- | --- | --- | --- | --- | --- | --- |
|  | Model 1 | | Model 2 | |  | Model 1 | | Model 2 | |  |
|  | HR (95%CI) | P value | HR (95%CI) | P value |  | HR (95%CI) | P value | HR (95%CI) | P value |  |
| **PM_2.5_ (μg/m3)** |  |  |  |  |  |  |  |  |  | 0.427 |
| Q1 | 1.00 (Ref) |  | 1.00 (Ref) |  |  | 1.00 (Ref) |  | 1.00 (Ref) |  |  |
| Q2 | 1.26 (0.92, 1.74) | 0.152 | 1.23 (0.89, 1.70) | 0.214 |  | 1.58 (1.04, 2.39) | 0.032 | 1.54 (1.02, 2.34) | 0.042 |  |
| Q3 | 1.38 (0.99, 1.92) | 0.055 | 1.28 (0.91, 1.79) | 0.155 |  | 1.47 (0.97, 2.22) | 0.068 | 1.44 (0.95, 2.20) | 0.089 |  |
| Q4 | 1.38 (1.01, 1.89) | 0.043 | 1.24 (0.89, 1.73) | 0.206 |  | 1.82 (1.21, 2.75) | 0.004 | 1.74 (1.13, 2.68) | 0.012 |  |
| P for trend |  | 0.042 |  | 0.230 |  |  | 0.007 |  | 0.018 |  |
| Per 5-μg/m3*day | 1.70 (1.08, 2.68) | 0.023 | 1.40 (0.85, 2.29) | 0.184 |  | 1.80 (0.98, 3.30) | 0.058 | 1.67 (0.86, 3.24) | 0.127 |  |
| **PM_10_ (μg/m3)** |  |  |  |  |  |  |  |  |  | 0.169 |
| Q1 | 1.00 (Ref) |  | 1.00 (Ref) |  |  | 1.00 (Ref) |  | 1.00 (Ref) |  |  |
| Q2 | 1.13 (0.82, 1.56) | 0.460 | 1.07 (0.77, 1.48) | 0.688 |  | 0.95 (0.65, 1.39) | 0.780 | 0.88 (0.60, 1.30) | 0.533 |  |
| Q3 | 1.22 (0.89, 1.67) | 0.213 | 1.11 (0.81, 1.53) | 0.518 |  | 0.90 (0.60, 1.34) | 0.593 | 0.86 (0.57, 1.29) | 0.471 |  |
| Q4 | 1.45 (1.07, 1.97) | 0.018 | 1.36 (0.99, 1.87) | 0.060 |  | 1.04 (0.71, 1.54) | 0.824 | 0.96 (0.64, 1.45) | 0.858 |  |
| P for trend |  | 0.015 |  | 0.053 |  |  | 0.851 |  | 0.870 |  |
| Per 5-μg/m3*day | 1.40 (1.06, 1.86) | 0.018 | 1.35 (1.00, 1.83) | 0.049 |  | 1.26 (0.89, 1.78) | 0.201 | 1.21 (0.83, 1.77) | 0.328 |  |
| **NO_2_ (μg/m3)** |  |  |  |  |  |  |  |  |  | 0.396 |
| Q1 | 1.00 (Ref) |  | 1.00 (Ref) |  |  | 1.00 (Ref) |  | 1.00 (Ref) |  |  |
| Q2 | 1.42 (1.01, 1.98) | 0.042 | 1.33 (0.95, 1.87) | 0.098 |  | 1.63 (1.09, 2.45) | 0.018 | 1.62 (1.08, 2.44) | 0.019 |  |
| Q3 | 1.61 (1.16, 2.23) | 0.004 | 1.53 (1.09, 2.14) | 0.013 |  | 1.54 (1.02, 2.32) | 0.039 | 1.48 (0.97, 2.24) | 0.067 |  |
| Q4 | 1.67 (1.21, 2.32) | 0.002 | 1.54 (1.09, 2.19) | 0.016 |  | 1.45 (0.96, 2.20) | 0.080 | 1.34 (0.85, 2.10) | 0.204 |  |
| P for trend |  | 0.002 |  | 0.017 |  |  | 0.137 |  | 0.278 |  |
| Per 5-μg/m3*day | 1.07 (1.01, 1.14) | 0.014 | 1.06 (1.00, 1.13) | 0.059 |  | 1.06 (0.99, 1.14) | 0.095 | 1.06 (0.97, 1.15) | 0.184 |  |
| **NO_X_ (μg/m3)** |  |  |  |  |  |  |  |  |  | 0.853 |
| Q1 | 1.00 (Ref) |  | 1.00 (Ref) |  |  | 1.00 (Ref) |  | 1.00 (Ref) |  |  |
| Q2 | 1.22 (0.88, 1.71) | 0.230 | 1.19 (0.85, 1.66) | 0.317 |  | 1.38 (0.92, 2.06) | 0.122 | 1.36 (0.91, 2.04) | 0.139 |  |
| Q3 | 1.35 (0.97, 1.86) | 0.074 | 1.28 (0.92, 1.79) | 0.147 |  | 1.43 (0.96, 2.14) | 0.078 | 1.34 (0.89, 2.03) | 0.159 |  |
| Q4 | 1.52 (1.11, 2.08) | 0.009 | 1.40 (1.00, 1.95) | 0.051 |  | 1.49 (0.99, 2.23) | 0.054 | 1.43 (0.93, 2.20) | 0.104 |  |
| P for trend |  | 0.007 |  | 0.048 |  |  | 0.054 |  | 0.115 |  |
| Per 5-μg/m3*day | 1.04 (1.01, 1.08) | 0.008 | 1.03 (1.00, 1.07) | 0.050 |  | 1.03 (0.99, 1.08) | 0.195 | 1.02 (0.97, 1.07) | 0.395 |  |
| **Combined air pollution exposure score (per SD)** | 1.16 (1.04, 1.29) | 0.008 | 1.13 (1.01, 1.28) | 0.040 |  | 1.11 (0.97, 1.27) | 0.118 | 1.09 (0.94, 1.27) | 0.266 | 0.632 |

Model 1 adjusted for age at recruitment and sex.

Model 2 adjusted for age at recruitment, sex, education, Townsend deprivation index (TDI), ethnicity, family history of CRC, BMI, alcohol intake and physical activity.

**Supplementary Table 14.** **Associations between individual and combined exposure to air pollution and risk of all-cause mortality in CRC survivors stratified by physical activity in UK Biobank.**

| Air pollutant | **Insufficient physical activity** | | | |  | **Sufficient physical activity** | | | | P interaction |
| --- | --- | --- | --- | --- | --- | --- | --- | --- | --- | --- |
|  | Model 1 | | Model 2 | |  | Model 1 | | Model 2 | |  |
|  | HR (95%CI) | P value | HR (95%CI) | P value |  | HR (95%CI) | P value | HR (95%CI) | P value |  |
| **PM_2.5_ (μg/m3)** |  |  |  |  |  |  |  |  |  | 0.152 |
| Q1 | 1.00 (Ref) |  | 1.00 (Ref) |  |  | 1.00 (Ref) |  | 1.00 (Ref) |  |  |
| Q2 | 1.45 (1.08, 1.94) | 0.013 | 1.38 (1.03, 1.85) | 0.031 |  | 1.13 (0.62, 2.09) | 0.684 | 1.22 (0.65, 2.31) | 0.532 |  |
| Q3 | 1.50 (1.12, 2.01) | 0.007 | 1.39 (1.03, 1.88) | 0.032 |  | 0.88 (0.45, 1.72) | 0.708 | 0.90 (0.45, 1.80) | 0.759 |  |
| Q4 | 1.69 (1.27, 2.24) | 0.000 | 1.52 (1.12, 2.04) | 0.006 |  | 1.03 (0.54, 1.97) | 0.922 | 1.20 (0.57, 2.51) | 0.626 |  |
| P for trend |  | 0.000 |  | 0.010 |  |  | 0.938 |  | 0.805 |  |
| Per 5-μg/m3*day | 1.81 (1.22, 2.66) | 0.003 | 1.52 (1.00, 2.32) | 0.052 |  | 1.15 (0.35, 3.78) | 0.820 | 1.41 (0.36, 5.45) | 0.620 |  |
| **PM_10_ (μg/m3)** |  |  |  |  |  |  |  |  |  | 0.969 |
| Q1 | 1.00 (Ref) |  | 1.00 (Ref) |  |  | 1.00 (Ref) |  | 1.00 (Ref) |  |  |
| Q2 | 1.03 (0.78, 1.35) | 0.850 | 0.97 (0.73, 1.29) | 0.842 |  | 1.15 (0.61, 2.16) | 0.665 | 1.33 (0.69, 2.55) | 0.394 |  |
| Q3 | 1.00 (0.76, 1.32) | 0.995 | 0.93 (0.71, 1.23) | 0.618 |  | 1.31 (0.68, 2.52) | 0.414 | 1.47 (0.74, 2.90) | 0.272 |  |
| Q4 | 1.21 (0.93, 1.57) | 0.164 | 1.10 (0.83, 1.45) | 0.502 |  | 1.10 (0.55, 2.19) | 0.782 | 1.27 (0.61, 2.65) | 0.518 |  |
| P for trend |  | 0.170 |  | 0.509 |  |  | 0.709 |  | 0.488 |  |
| Per 5-μg/m3*day | 1.30 (1.02, 1.65) | 0.032 | 1.21 (0.94, 1.57) | 0.145 |  | 1.20 (0.64, 2.26) | 0.573 | 1.34 (0.68, 2.67) | 0.398 |  |
| **NO_2_ (μg/m3)** |  |  |  |  |  |  |  |  |  | 0.547 |
| Q1 | 1.00 (Ref) |  | 1.00 (Ref) |  |  | 1.00 (Ref) |  | 1.00 (Ref) |  |  |
| Q2 | 1.51 (1.13, 2.01) | 0.006 | 1.44 (1.08, 1.94) | 0.014 |  | 1.10 (0.55, 2.18) | 0.794 | 1.17 (0.58, 2.36) | 0.666 |  |
| Q3 | 1.49 (1.11, 1.99) | 0.007 | 1.39 (1.04, 1.88) | 0.029 |  | 1.92 (1.04, 3.56) | 0.039 | 2.09 (1.10, 3.98) | 0.024 |  |
| Q4 | 1.75 (1.32, 2.32) | 0.000 | 1.61 (1.19, 2.18) | 0.002 |  | 1.03 (0.50, 2.12) | 0.934 | 1.11 (0.51, 2.45) | 0.791 |  |
| P for trend |  | 0.000 |  | 0.005 |  |  | 0.574 |  | 0.448 |  |
| Per 5-μg/m3*day | 1.08 (1.03, 1.13) | 0.002 | 1.07 (1.01, 1.13) | 0.023 |  | 1.02 (0.89, 1.17) | 0.778 | 1.05 (0.89, 1.23) | 0.563 |  |
| **NO_X_ (μg/m3)** |  |  |  |  |  |  |  |  |  | 0.088 |
| Q1 | 1.00 (Ref) |  | 1.00 (Ref) |  |  | 1.00 (Ref) |  | 1.00 (Ref) |  |  |
| Q2 | 1.33 (0.99, 1.78) | 0.060 | 1.30 (0.97, 1.74) | 0.084 |  | 0.90 (0.48, 1.70) | 0.751 | 0.97 (0.51, 1.86) | 0.926 |  |
| Q3 | 1.46 (1.10, 1.95) | 0.010 | 1.37 (1.02, 1.84) | 0.038 |  | 1.10 (0.59, 2.05) | 0.768 | 1.23 (0.63, 2.37) | 0.547 |  |
| Q4 | 1.73 (1.30, 2.28) | 0.000 | 1.59 (1.18, 2.14) | 0.002 |  | 0.87 (0.45, 1.69) | 0.681 | 0.95 (0.46, 1.97) | 0.882 |  |
| P for trend |  | 0.000 |  | 0.003 |  |  | 0.814 |  | 0.972 |  |
| Per 5-μg/m3*day | 1.04 (1.01, 1.07) | 0.003 | 1.03 (1.00, 1.06) | 0.029 |  | 1.00 (0.91, 1.09) | 0.926 | 1.02 (0.92, 1.13) | 0.698 |  |
| **Combined air pollution exposure score (per SD)** | 1.15 (1.05, 1.26) | 0.003 | 1.12 (1.01, 1.24) | 0.025 |  | 1.04 (0.81, 1.33) | 0.754 | 1.11 (0.83, 1.48) | 0.474 | 0.411 |

Model 1 adjusted for age at recruitment and sex.

Model 2 adjusted for age at recruitment, sex, education, Townsend deprivation index (TDI), ethnicity, family history of CRC, BMI, alcohol intake, and smoking.

* Sufficient physical activity was defined by at least ≥150 minutes moderate activity per week or ≥75 minutes vigorous activity per week (or an equivalent combination) according to the 2018 Physical Activity Guidelines for Americans (DOI: 10.1001/jama.2018.14854).

**Supplementary Table 15.** **Associations between individual and combined exposure to air pollution and risk of all-cause mortality in CRC survivors stratified by anatomic sites in UK Biobank.**

| Air pollutant | **Colon** | | | |  | **Rectum** | | | |
| --- | --- | --- | --- | --- | --- | --- | --- | --- | --- |
|  | Model 1 | | Model 2 | |  | Model 1 | | Model 2 | |
|  | HR (95%CI) | P value | HR (95%CI) | P value |  | HR (95%CI) | P value | HR (95%CI) | P value |
| **PM_2.5_ (μg/m3)** |  |  |  |  |  |  |  |  |  |
| Q1 | 1.00 (Ref) |  | 1.00 (Ref) |  |  | 1.00 (Ref) |  | 1.00 (Ref) |  |
| Q2 | 1.27 (0.91, 1.76) | 0.164 | 1.26 (0.90, 1.76) | 0.171 |  | 1.98 (1.20, 3.28) | 0.007 | 1.65 (0.99, 2.76) | 0.054 |
| Q3 | 1.36 (0.97, 1.90) | 0.072 | 1.33 (0.94, 1.87) | 0.107 |  | 1.92 (1.16, 3.18) | 0.011 | 1.56 (0.93, 2.63) | 0.092 |
| Q4 | 1.46 (1.05, 2.03) | 0.024 | 1.46 (1.03, 2.08) | 0.034 |  | 1.96 (1.18, 3.25) | 0.009 | 1.50 (0.88, 2.57) | 0.139 |
| P for trend |  | 0.022 |  | 0.034 |  |  | 0.014 |  | 0.222 |
| Per 5-μg/m3*day | 1.74 (1.05, 2.89) | 0.033 | 1.68 (0.97, 2.90) | 0.062 |  | 3.02 (1.46, 6.24) | 0.003 | 1.94 (0.87, 4.32) | 0.107 |
| **PM_10_ (μg/m3)** |  |  |  |  |  |  |  |  |  |
| Q1 | 1.00 (Ref) |  | 1.00 (Ref) |  |  | 1.00 (Ref) |  | 1.00 (Ref) |  |
| Q2 | 0.98 (0.71, 1.36) | 0.904 | 0.97 (0.70, 1.35) | 0.874 |  | 1.39 (0.88, 2.21) | 0.162 | 1.23 (0.77, 1.96) | 0.396 |
| Q3 | 1.23 (0.89, 1.68) | 0.205 | 1.20 (0.87, 1.66) | 0.270 |  | 1.07 (0.65, 1.76) | 0.793 | 0.87 (0.52, 1.45) | 0.602 |
| Q4 | 1.08 (0.78, 1.50) | 0.627 | 1.07 (0.76, 1.50) | 0.701 |  | 1.75 (1.11, 2.76) | 0.015 | 1.37 (0.84, 2.21) | 0.203 |
| P for trend |  | 0.431 |  | 0.521 |  |  | 0.030 |  | 0.324 |
| Per 5-μg/m3*day | 1.27 (0.95, 1.70) | 0.113 | 1.27 (0.93, 1.75) | 0.132 |  | 1.62 (1.08, 2.43) | 0.021 | 1.33 (0.85, 2.09) | 0.211 |
| **NO_2_ (μg/m3)** |  |  |  |  |  |  |  |  |  |
| Q1 | 1.00 (Ref) |  | 1.00 (Ref) |  |  | 1.00 (Ref) |  | 1.00 (Ref) |  |
| Q2 | 1.37 (0.98, 1.91) | 0.069 | 1.34 (0.96, 1.89) | 0.089 |  | 2.06 (1.25, 3.39) | 0.005 | 1.76 (1.06, 2.92) | 0.029 |
| Q3 | 1.49 (1.08, 2.07) | 0.016 | 1.50 (1.07, 2.09) | 0.018 |  | 1.81 (1.09, 3.02) | 0.023 | 1.45 (0.86, 2.45) | 0.168 |
| Q4 | 1.48 (1.06, 2.07) | 0.023 | 1.47 (1.02, 2.11) | 0.038 |  | 2.16 (1.31, 3.56) | 0.003 | 1.64 (0.96, 2.82) | 0.073 |
| P for trend |  | 0.024 |  | 0.036 |  |  | 0.008 |  | 0.172 |
| Per 5-μg/m3*day | 1.08 (1.01, 1.15) | 0.015 | 1.08 (1.01, 1.16) | 0.018 |  | 1.08 (0.99, 1.18) | 0.073 | 1.04 (0.94, 1.15) | 0.501 |
| **NO_X_ (μg/m3)** |  |  |  |  |  |  |  |  |  |
| Q1 | 1.00 (Ref) |  | 1.00 (Ref) |  |  | 1.00 (Ref) |  | 1.00 (Ref) |  |
| Q2 | 1.02 (0.73, 1.43) | 0.906 | 1.02 (0.72, 1.44) | 0.907 |  | 1.92 (1.18, 3.13) | 0.009 | 1.74 (1.06, 2.85) | 0.028 |
| Q3 | 1.41 (1.02, 1.94) | 0.039 | 1.39 (1.00, 1.94) | 0.051 |  | 1.58 (0.95, 2.62) | 0.077 | 1.29 (0.76, 2.17) | 0.344 |
| Q4 | 1.44 (1.04, 1.98) | 0.026 | 1.47 (1.04, 2.08) | 0.028 |  | 1.94 (1.18, 3.19) | 0.009 | 1.45 (0.85, 2.45) | 0.173 |
| P for trend | 1.01 (1.00, 1.03) | 0.008 | 1.02 (1.00, 1.03) | 0.011 |  | 1.02 (1.00, 1.03) | 0.025 | 1.01 (0.99, 1.02) | 0.360 |
| Per 5-μg/m3*day |  | 0.008 |  | 0.012 |  |  | 0.047 |  | 0.483 |
| **Combined air pollution exposure score (per SD)** | 1.14 (1.02, 1.28) | 0.019 | 1.14 (1.04, 1.25) | 0.032 |  | 1.19 (1.01, 1.39) | 0.034 | 1.10 (0.92, 1.33) | 0.298 |

Model 1 adjusted for age at recruitment and sex.

Model 2 adjusted for age at recruitment, sex, education, Townsend deprivation index (TDI), ethnicity, family history of CRC, BMI, alcohol intake, and smoking.

* Sufficient physical activity was defined by at least ≥150 minutes moderate activity per week or ≥75 minutes vigorous activity per week (or an equivalent combination) according to the 2018 Physical Activity Guidelines for Americans (DOI: 10.1001/jama.2018.14854).

**Supplementary Table 16.** **Articles and reported effect estimates included in the meta-analysis of PM exposure and CRC.**

| **Article** | **Study period** | **Published year** | **Location** | **Measure of association** | **Cancer site** | **Outcome** | **Outcome estimate^a^** | **95% CI** |
| --- | --- | --- | --- | --- | --- | --- | --- | --- |
| Turner et al. | 1982-2004 | 2017 | USA | HR per 4.4 μg/m3 PM_2.5_ | CRC | Mortality | 1.09 (1.04)^b^ | 1.00, 1.19 (1.00, 1.08)^b^ |
| Chu et al. | 1993-2001 | 2021 | USA | HR per 5 μg/m3 PM_2.5_ | CRC | Incidence | 2.40 (1.55)^b^ | 1.95, 2.96 (1.40, 1.72)^b^ |
| Coleman et al. | 1987-2014 | 2020 | USA | HR per 10 μg/m3 PM_2.5_ | CRC | Mortality | 1.29 | 1.05, 1.58 |
| Guo et al. | 2001-2016 | 2020 | Taiwan | HR per 10 μg/m3 PM_2.5_ | CRC | Mortality | 1.13 | 1.00, 1.26 |
| Pei et al. | 2010-2016 | 2022 | Brazil | HR per 1 μg/m3 PM_2.5_ | CRC | Mortality | 2.16 (1.08)^b^ | 1.63, 2.86 (1.05, 1.11)^b^ |
| Shin et al. | 2007-2015 | 2022 | South Korea | HR per 10 μg/m3 PM_2.5_ | CRC | Mortality | 0.88 | 0.26, 3.02 |

^a.^ Fully adjusted outcome estimates reported as available from included individual articles.

^b.^ Standardized to a 10 μg/m3 increase in PM_2.5_ (original values included in parenthesis).

**Supplementary Table 17. PM_2.5_-related CpG sites and mQTLs from GoDMC database.** (details in Supplementary file2)

**Supplementary Table 18. PM_10_-related CpG sites and mQTLs from GoDMC database.** (details in Supplementary file2)

**Supplementary Table 19. NO_2_-related CpG sites and mQTLs from GoDMC database.** (details in Supplementary file2)

**Supplementary Table 20. Dose-response effect of combined air pollution on the risk of CRC incidence stratified by the genotype of rs1870123, rs876961,rs497916 and rs11708390 in the UK Biobank.**

| **CpG mQTL** | **Genotype status** | **cases** | **APES (per SD)** | | | | |
| --- | --- | --- | --- | --- | --- | --- | --- |
|  |  |  | **Model 1** | |  | **Model 2** | |
|  |  |  | **RR (95% CI)** | **P value** |  | **RR (95% CI)** | **P value** |
| rs1870123 | GG | 724 | 1.03 (0.96, 1.11) | 0.425 |  | 1.03 (0.95, 1.11) | 0.456 |
|  | GA | 2584 | 1.00 (0.96, 1.04) | 0.924 |  | 1.01 (0.97, 1.05) | 0.644 |
|  | AA | 2284 | 1.07 (1.02, 1.12) | 0.003 |  | 1.07 (1.02, 1.12) | 0.006 |
| rs876961 | GG | 1927 | 1.04 (0.99, 1.09) | 0.143 |  | 1.04 (0.99, 1.09) | 0.099 |
|  | GA | 2677 | 1.04 (1.00, 1.08) | 0.060 |  | 1.04 (1.00, 1.08) | 0.069 |
|  | AA | 988 | 1.00 (0.94, 1.07) | 0.932 |  | 1.01 (0.94, 1.07) | 0.866 |
| rs497916 | TT | 423 | 1.03 (0.93, 1.14) | 0.575 |  | 1.02 (0.93, 1.13) | 0.671 |
|  | TC | 2239 | 1.07 (1.02, 1.12) | 0.003 |  | 1.08 (1.03, 1.13) | 0.002 |
|  | CC | 2930 | 1.00 (0.97, 1.04) | 0.805 |  | 1.01 (0.97, 1.05) | 0.737 |
| rs11708390 | AA | 103 | 1.06 (0.85, 1.31) | 0.621 |  | 1.07 (0.87, 1.33) | 0.520 |
|  | AC | 1319 | 1.03 (0.97, 1.09) | 0.362 |  | 1.02 (0.97, 1.08) | 0.418 |
|  | CC | 4170 | 1.03 (1.00, 1.07) | 0.049 |  | 1.04 (1.00, 1.07) | 0.031 |

Model 1 adjusted for age at recruitment and sex.

Model 2 adjusted for age at recruitment, sex, education, TDI, ethnicity, family history of CRC, BMI, alcohol intake, physical activity, smoking and first 10 genetic principal components.

**Supplementary Table 21. Dose-response effect of individual or combined air pollution on the CRC survival stratified by the genotype of rs1870123, rs876961,rs497916 and rs11708390 in the UK Biobank.**

| **CpG mQTL** | **Genotype status** | **cases** | **APES (per SD)** | | | | | **Log-rank  P** |  | **PM_2.5_ (5-μg/m3*day)** | | | | | **Log-rank  P** |  | **NO_2_ (5-μg/m3*day)** | | | | | **Log-rank  P** |
| --- | --- | --- | --- | --- | --- | --- | --- | --- | --- | --- | --- | --- | --- | --- | --- | --- | --- | --- | --- | --- | --- | --- |
|  |  |  | **Model 1** | |  | **Model 2** | |  |  | **Model 1** | |  | **Model 2** | |  |  | **Model 1** | |  | **Model 2** | |  |
|  |  |  | **RR (95% CI)** | **P** |  | **RR (95% CI)** | **P** |  |  | **RR (95% CI)** | **P** |  | **RR (95% CI)** | **P** |  |  | **RR (95% CI)** | **P** |  | **RR (95% CI)** | **P** |  |
| rs1870123 | GG | 59 | 1.48 (1.12, 1.95) | 0.006 |  | 1.43 (1.05, 1.94) | 0.022 | 0.030 |  |  |  |  |  |  |  |  |  |  |  |  |  |  |
|  | GA | 225 | 1.02 (0.90, 1.17) | 0.715 |  | 0.99 (0.85, 1.14) | 0.841 | 0.979 |  |  |  |  |  |  |  |  |  |  |  |  |  |  |
|  | AA | 223 | 1.20 (1.06, 1.36) | 0.004 |  | 1.19 (1.04, 1.36) | 0.014 | 0.002 |  |  |  |  |  |  |  |  |  |  |  |  |  |  |
| rs876961 | GG | 173 | 1.06 (0.91, 1.25) | 0.450 |  | 1.07 (0.90, 1.27) | 0.474 | 0.376 |  | 0.95 (0.48, 1.90) | 0.894 |  | 0.90 (0.43, 1.88) | 0.785 | 0.208 |  |  |  |  |  |  |  |
|  | GA | 252 | 1.25 (1.11, 1.40) | 0.000 |  | 1.19 (1.05, 1.36) | 0.008 | 0.002 |  | 2.74 (1.64, 4.55) | 0.000 |  | 2.12 (1.22, 3.68) | 0.008 | 0.000 |  |  |  |  |  |  |  |
|  | AA | 82 | 1.00 (0.81, 1.24) | 0.966 |  | 1.01 (0.80, 1.29) | 0.907 | 0.875 |  | 1.31 (0.47, 3.60) | 0.605 |  | 1.24 (0.41, 3.71) | 0.702 | 0.240 |  |  |  |  |  |  |  |
| rs497916 | TT | 39 | 0.92 (0.65, 1.28) | 0.608 |  | 0.68 (0.43, 1.07) | 0.093 | 0.982 |  |  |  |  |  |  |  |  |  |  |  |  |  |  |
|  | TC | 185 | 1.35 (1.18, 1.54) | <0.001 |  | 1.34 (1.16, 1.55) | <0.001 | <0.001 |  |  |  |  |  |  |  |  |  |  |  |  |  |  |
|  | CC | 283 | 1.04 (0.92, 1.17) | 0.527 |  | 1.00 (0.88, 1.14) | 0.987 | 0.699 |  |  |  |  |  |  |  |  |  |  |  |  |  |  |
| rs11708390 | AA | 11 | 1.52 (0.55, 4.21) | 0.417 |  | 4.59 (0.92, 22.90) | 0.063 | 0.108 |  |  |  |  |  |  |  |  | 1.25 (0.68, 2.31) | 0.476 |  | 2.30 (0.83, 6.34) | 0.107 | 0.195 |
|  | AC | 115 | 1.13 (0.93, 1.36) | 0.208 |  | 1.07 (0.87, 1.32) | 0.532 | 0.953 |  |  |  |  |  |  |  |  | 1.08 (0.97, 1.20) | 0.158 |  | 1.05 (0.94, 1.18) | 0.404 | 0.427 |
|  | CC | 381 | 1.15 (1.04, 1.27) | 0.005 |  | 1.11 (1.00, 1.24) | 0.050 | 0.006 |  |  |  |  |  |  |  |  | 1.07 (1.01, 1.13) | 0.013 |  | 1.05 (0.99, 1.12) | 0.087 | 0.019 |

Model 1 adjusted for age at recruitment and sex.

Model 2 adjusted for age at recruitment, sex, education, TDI, ethnicity, family history of CRC, BMI, alcohol intake, physical activity, smoking, and first 10 genetic principal components.

**Supplementary Figures**


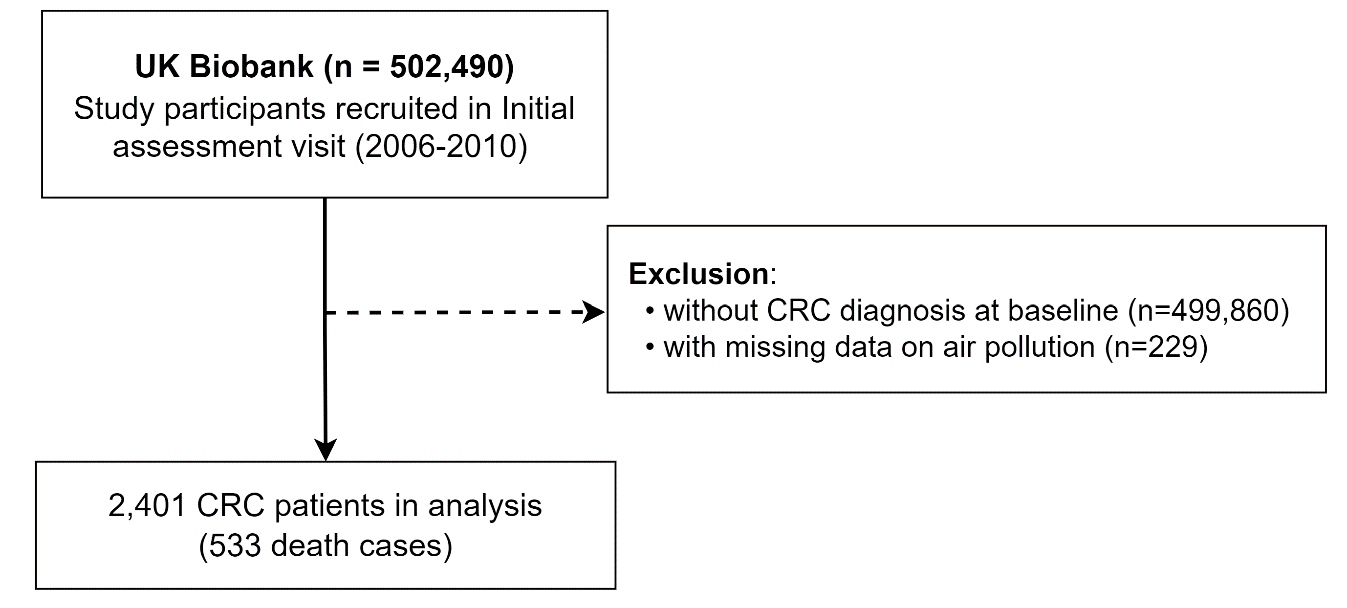

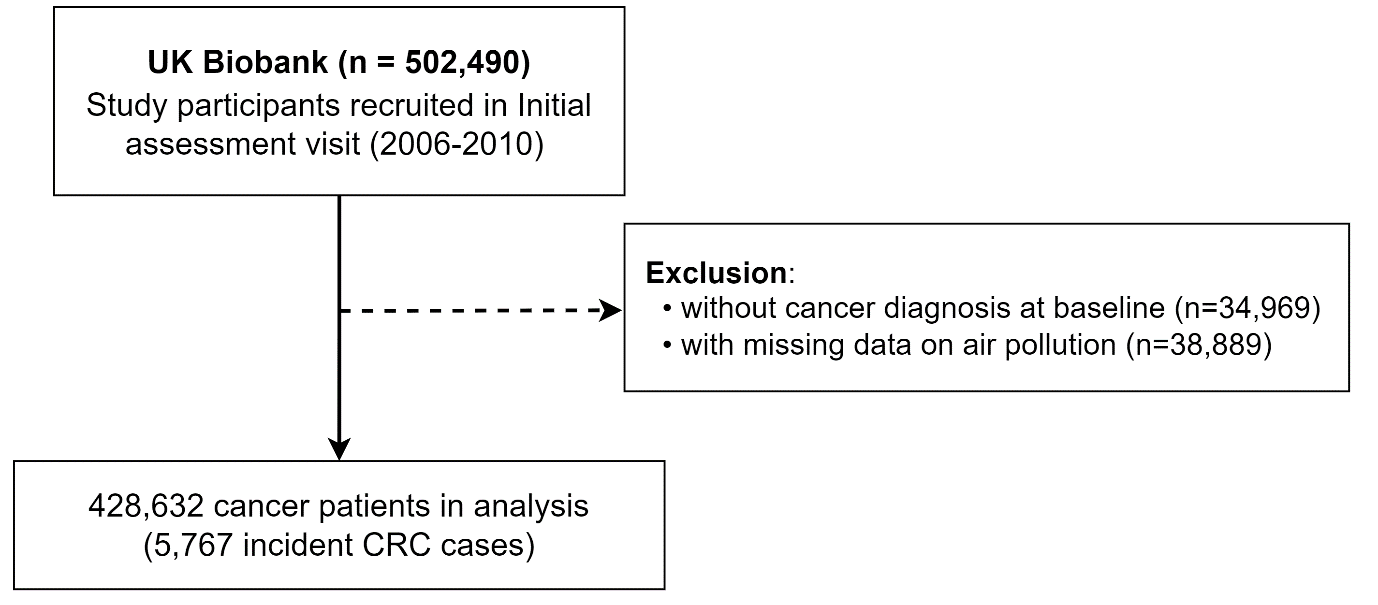


**(a) flowchart for CRC incidence analysis**

**(b) flowchart for CRC survival analysis**

**Supplementary Figure 1. Flowchart of population selection in the prospective cohort study.** (a) for CRC incidence analysis; (b) for overall survival analysis among CRC patients and survivors.


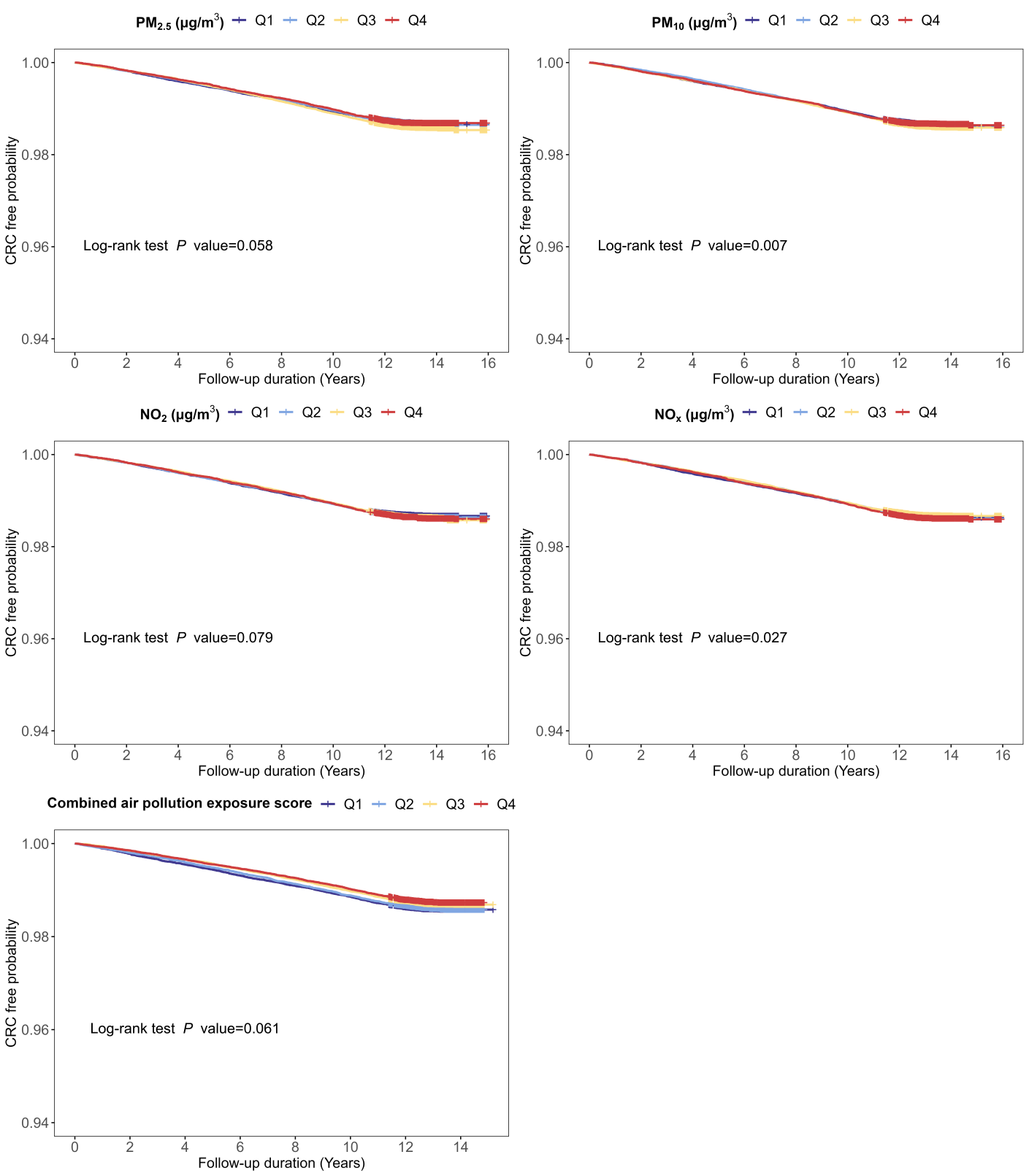


**Supplementary Figure 2. Kaplan-Meier analysis for long-term impact of air pollution on CRC incidence in the UK Biobank.** The adjusted Kaplan-Meier curves depicted CRC-free survival probability. The adjustments were recruitment, sex, education, Townsend deprivation index (TDI), ethnicity, family history of CRC, BMI, alcohol intake, physical activity and smoking.

**
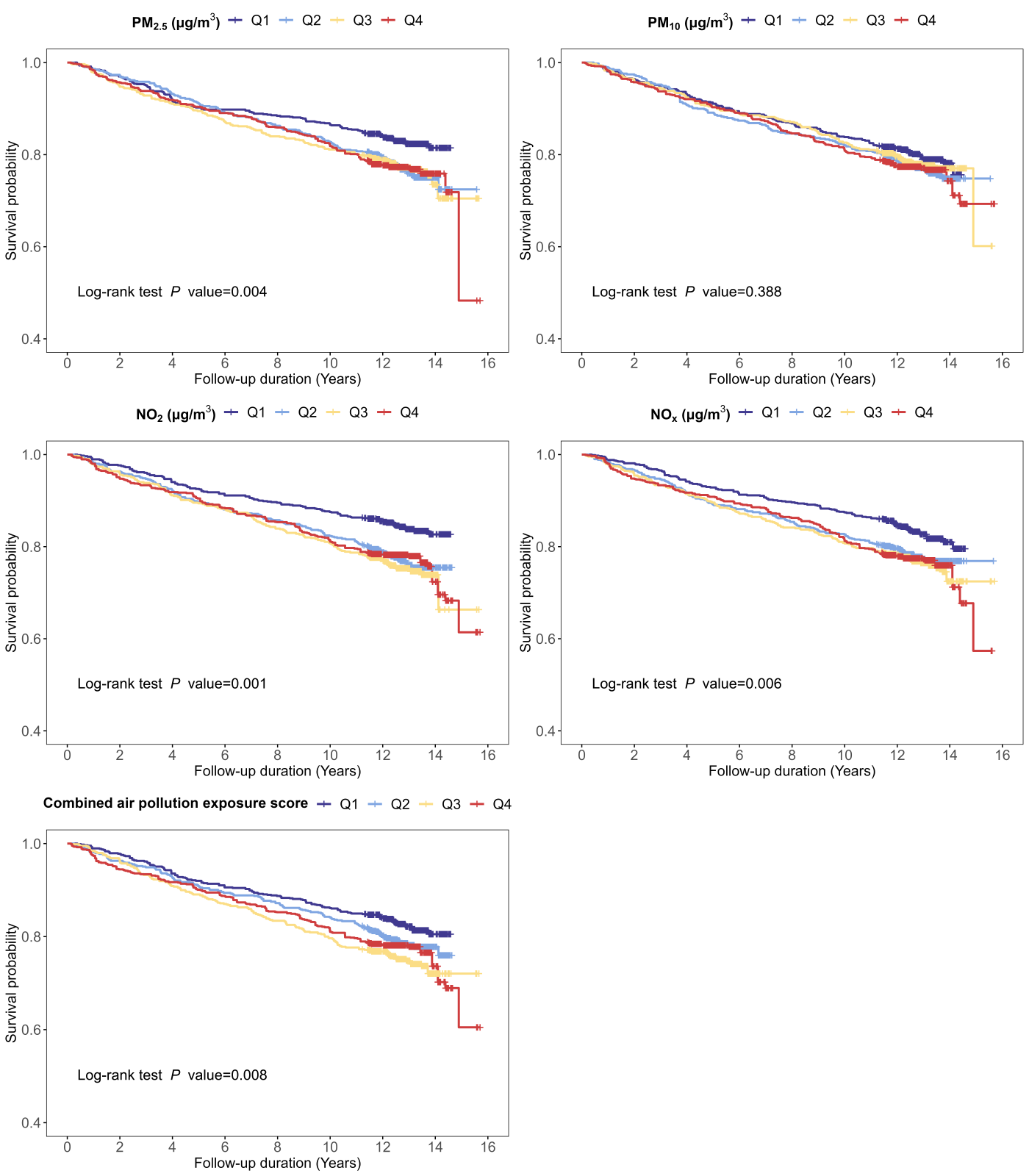
**

**Supplementary Figure 3. Kaplan-Meier analysis for long-term impact of air pollution on CRC survival in the UK Biobank.** The adjusted Kaplan-Meier curves depicted survival probability among CRC patients. The adjustments were recruitment, sex, education, Townsend deprivation index (TDI), ethnicity, family history of CRC, BMI, alcohol intake, physical activity and smoking.


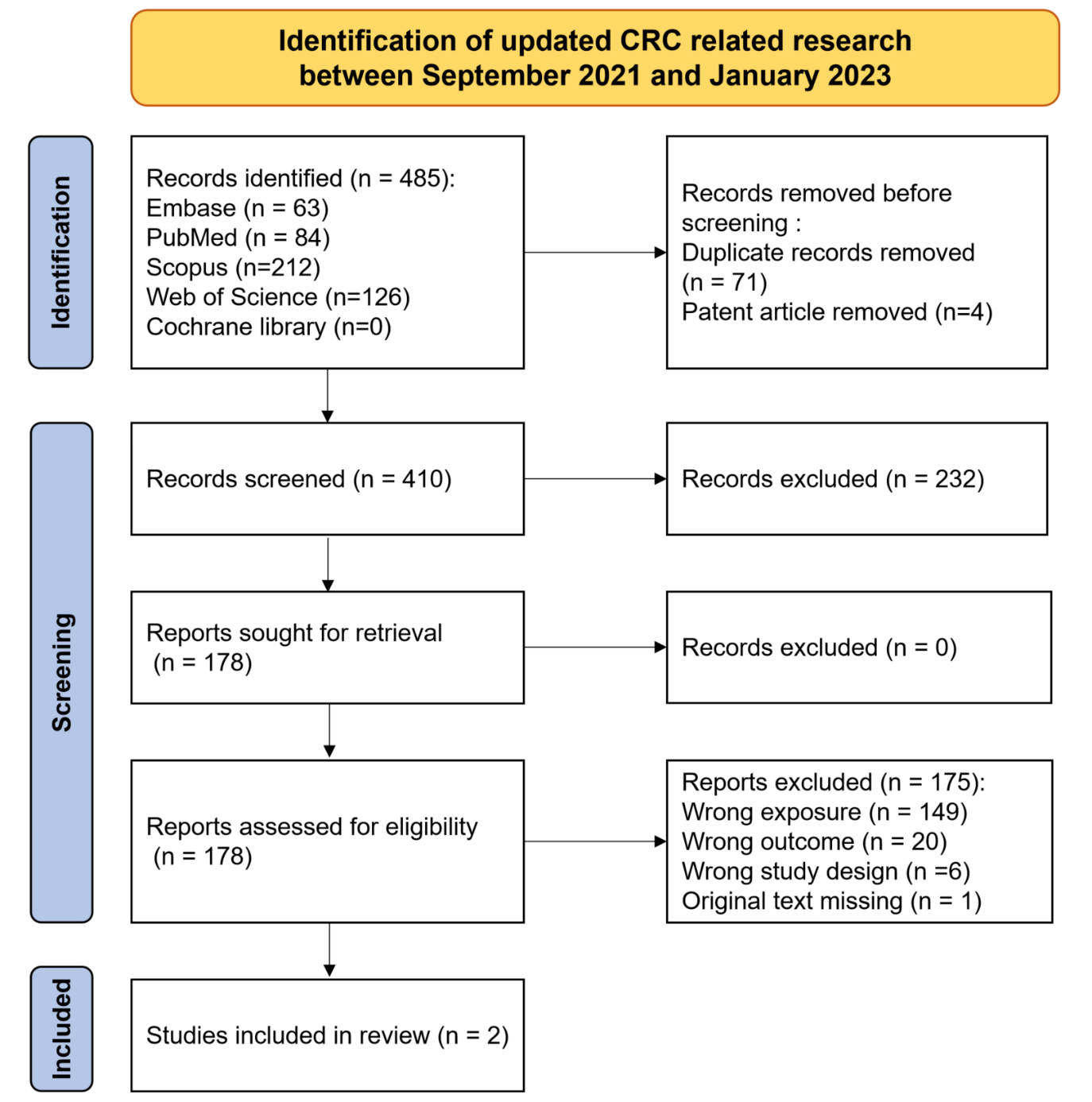


**Supplementary Figure 4. PRISMA flow diagram showing the literature search and screening process for studies relevant to PM exposure and CRC outcomes (published between September 2021 and January 2023).** PM, particulate matter; PRISMA, Preferred Reporting Items for Systematic Reviews and Meta-Analyses.


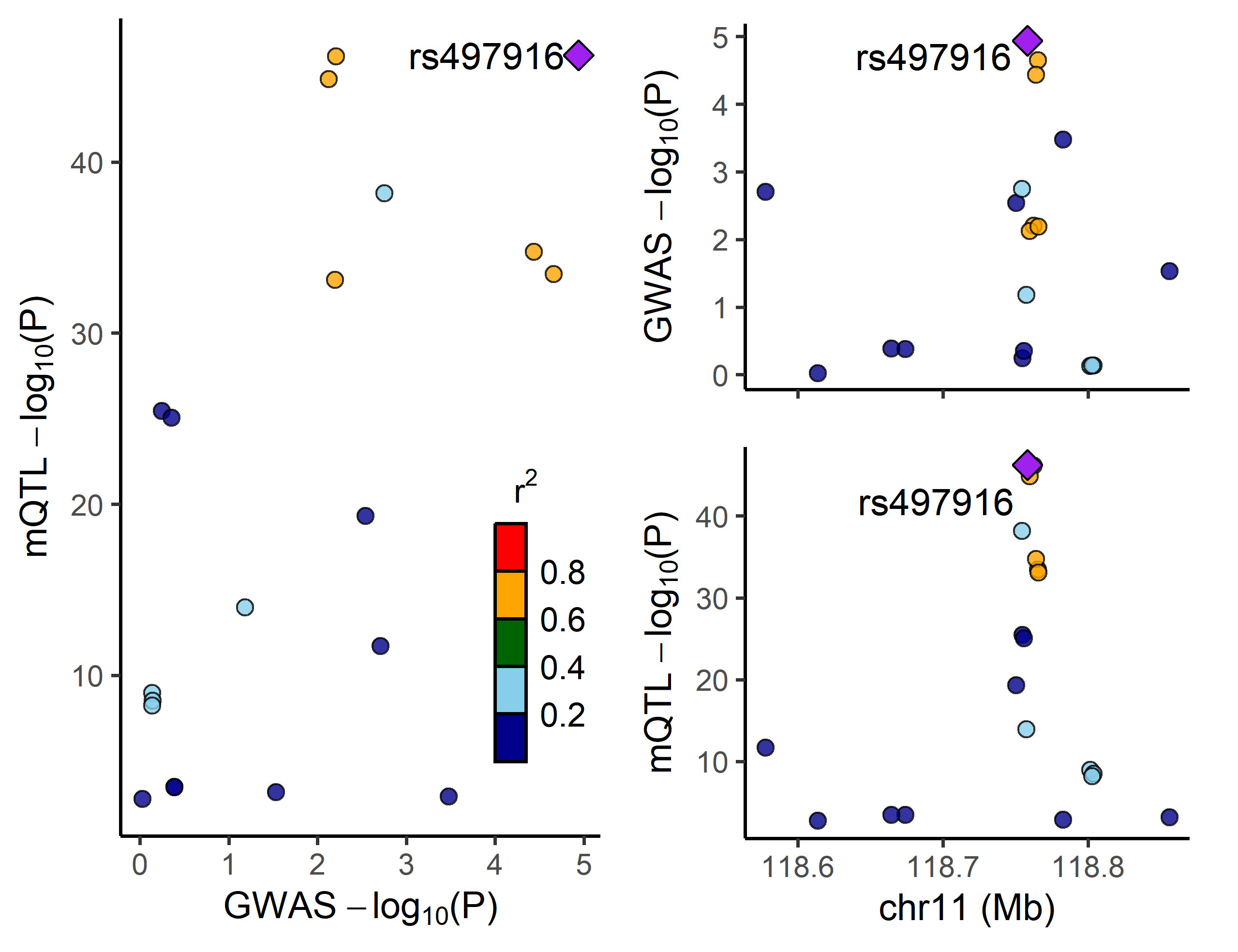


**Supplementary Figure 5. Regional plot for colocalization of methylation at cg16235962 [CXCR5] and CRC susceptibility (the posterior probability of mQTL rs497916 is 99.7%).**


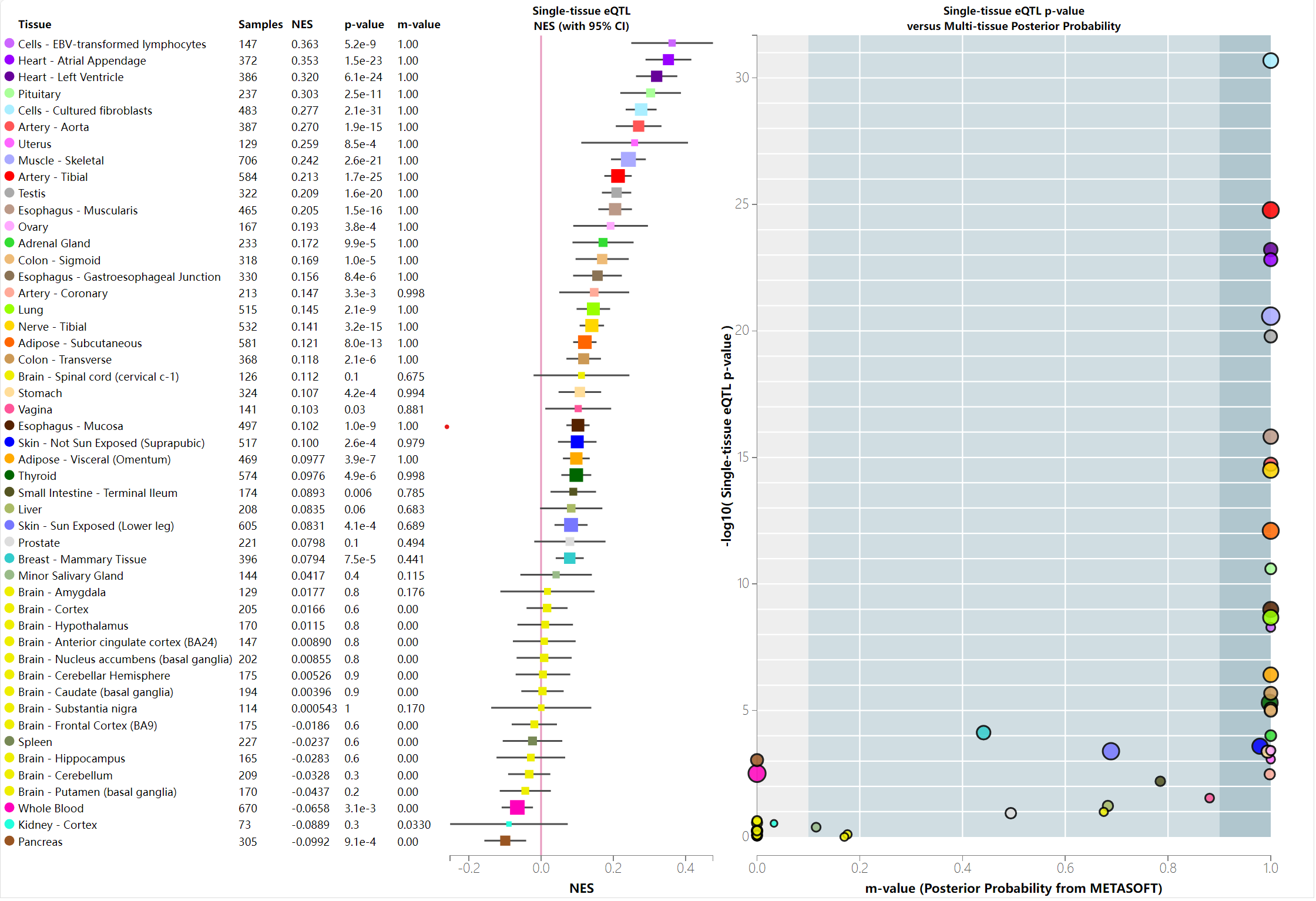


**Supplementary Figure 6. Single-tissue eQTL plot of rs1870123 (cg13835894, TMBIM1).** NES, normalized effect size. m value, the posterior probability that an eQTL effect exists in each tissue tested in the cross-tissue meta-analysis. Small m value (e.g., <0.1), the tissue is predicted to NOT have an eQTL effect; large m value (e.g., >0.9), the tissue is predicted to have an eQTL effect; otherwise, the prediction of the existence of an eQTL effect is ambiguous.


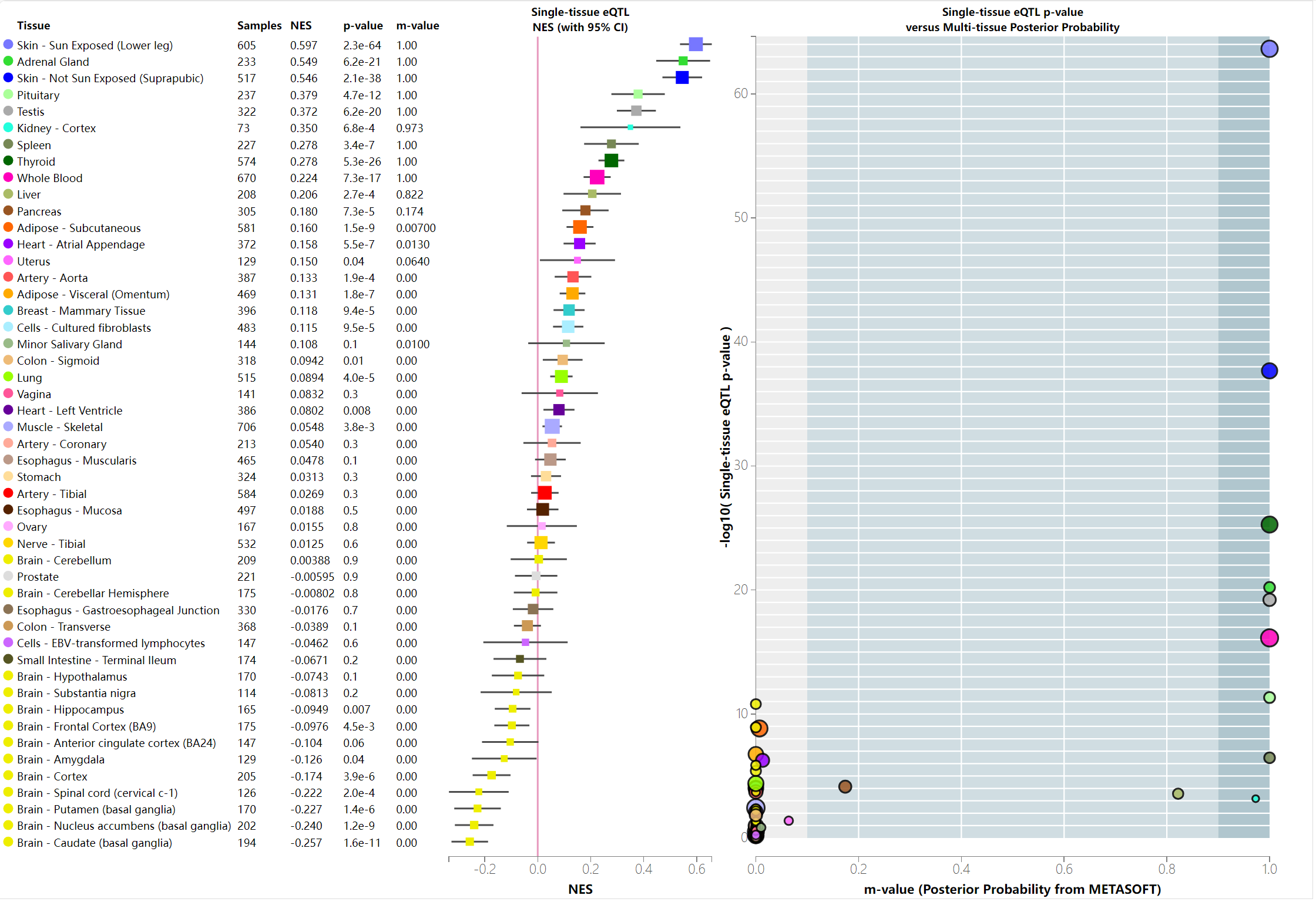
**Supplementary Figure 7. Single-tissue eQTL plot of rs1870123 (cg13835894, PNKD).** NES, normalized effect size. m value, the posterior probability that an eQTL effect exists in each tissue tested in the cross-tissue meta-analysis. Small m value (e.g., <0.1), the tissue is predicted to NOT have an eQTL effect; large m value (e.g., >0.9), the tissue is predicted to have an eQTL effect; otherwise, the prediction of the existence of an eQTL effect is ambiguous.


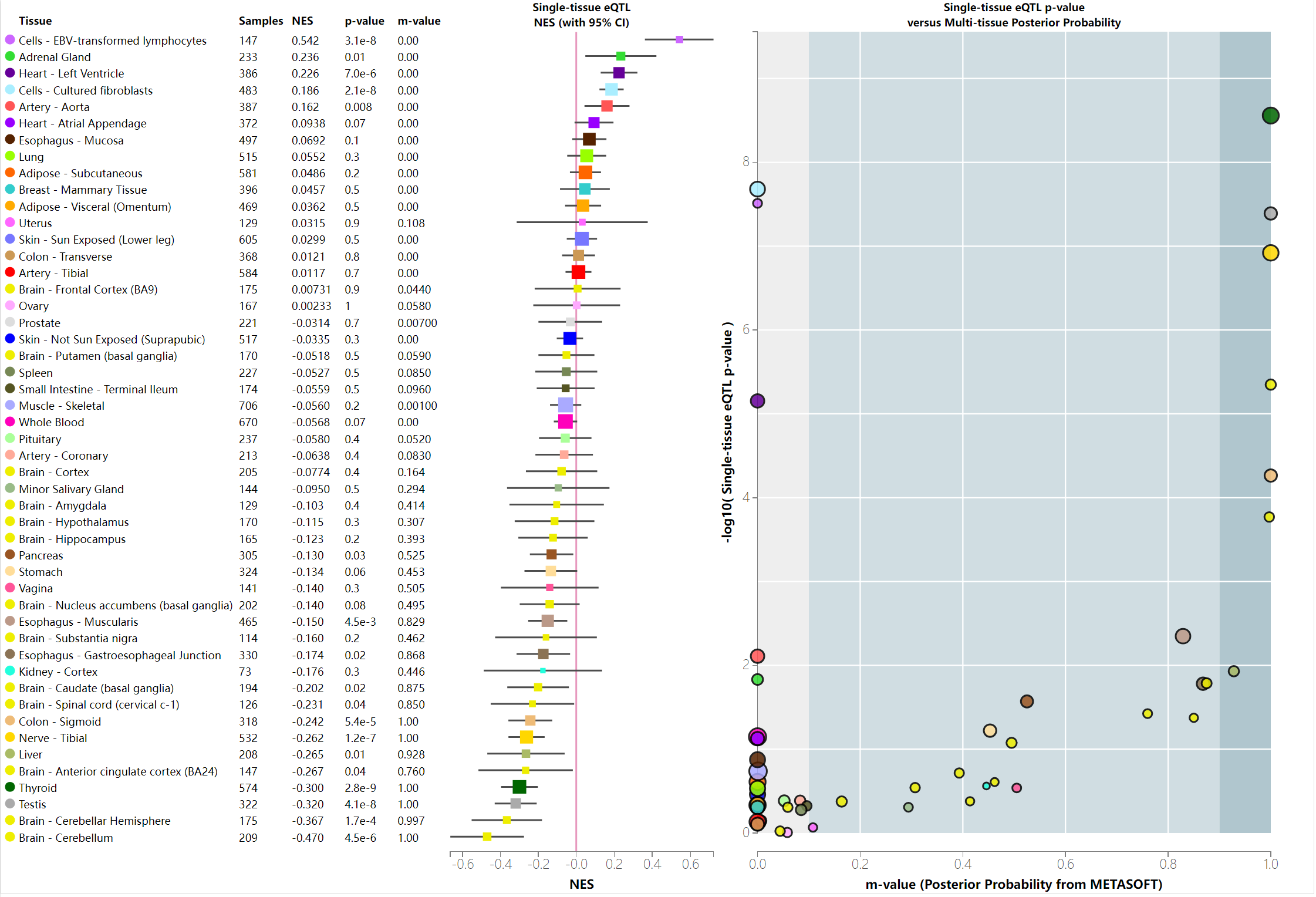


**Supplementary Figure 8. Single-tissue eQTL plot of rs11708390 (cg16947394, TMEM110).** NES, normalized effect size. m value, the posterior probability that an eQTL effect exists in each tissue tested in the cross-tissue meta-analysis. Small m value (e.g., <0.1), the tissue is predicted to NOT have an eQTL effect; large m value (e.g., >0.9), the tissue is predicted to have an eQTL effect; otherwise, the prediction of the existence of an eQTL effect is ambiguous.
